# Supplementary material for: Aversive memory formation in humans involves an amygdala-hippocampus phase code
Source: Nat Commun. 2022 Oct 27;13:6403. doi: 10.1038/s41467-022-33828-2 (PMC9613775; doi:10.1038/s41467-022-33828-2)
Supplement: Supplementary file 1 — Supplementary Information [file 41467_2022_33828_MOESM1_ESM.pdf]

Aversive memory formation in humans involves an amygdala-hippocampus phase code

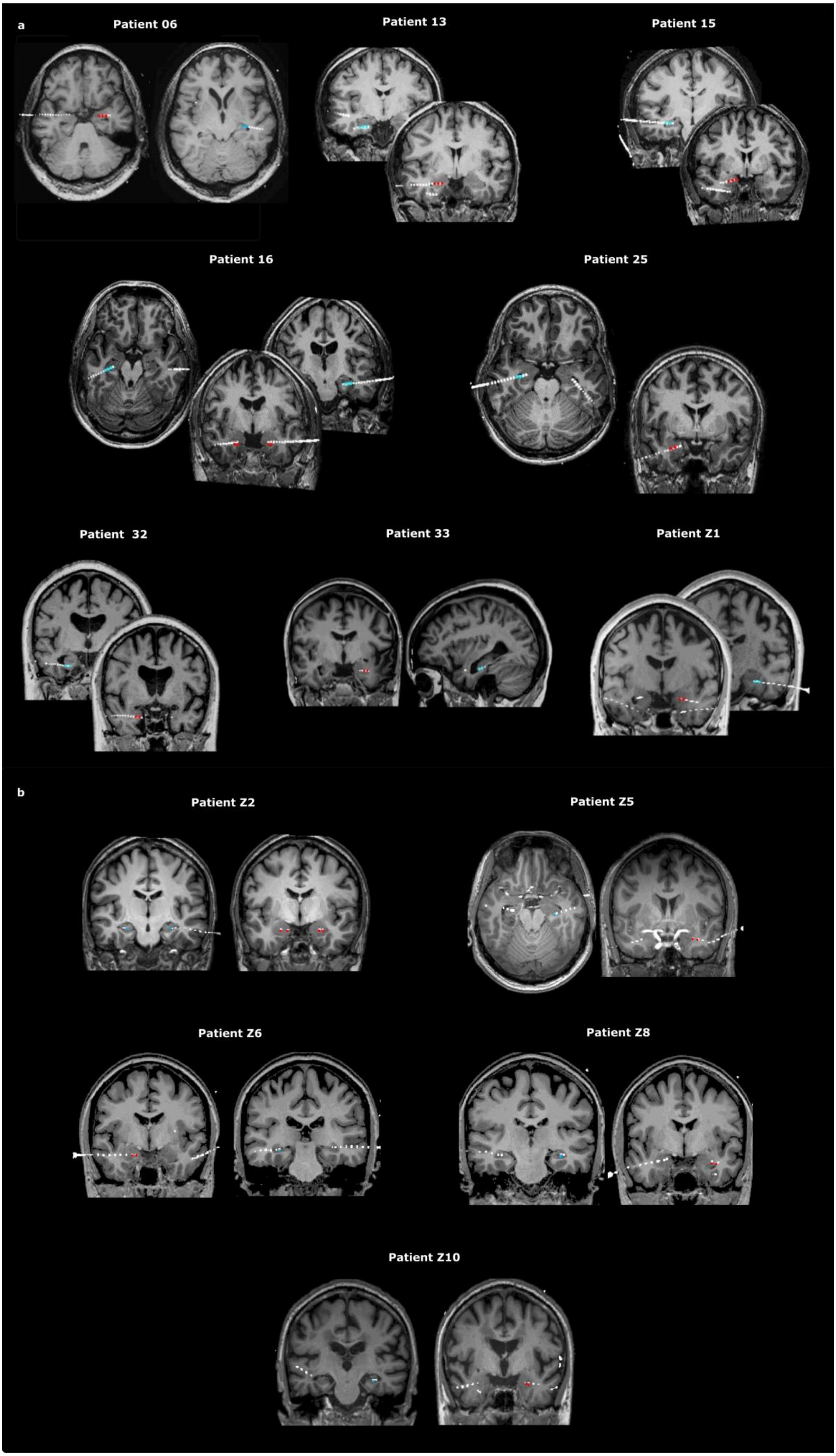
a
Patient 06
Patient 13
Patient 15
Patient 16
Patient 25
Patient 32
Patient 33
Patient Z1
b
Patient Z2
Patient Z5
Patient Z6
Patient Z8
Patient Z10

Supplementary Fig. 1. a, Electrode contact localization for the 8 patients (Cohort 1) with macro-electrodes localized in both amygdala and hippocampus. Coronal sections are shown for all patients; sagittal view is shown for patient 33, and axial sections for patient 6, 16 and 25. Red dots indicate amygdala contacts and blues dots hippocampal contacts included in our analyses. **b, Electrode contact localization for patients (Cohort 2) with macro-electrodes localized in both amygdala and hippocampus.** Coronal sections are shown for all patients; and axial sections for patient Z5, for better visualization of hippocampal contacts. Red dots indicate amygdala contacts. The blue dot represents the first hippocampal contact and point at putative microelectrode location. For all patients, post-operative CT images from each patient have been co-registered with their corresponding pre-operative MRI scans in native space and superimposed to display amygdala and hippocampal contacts (CTs have been thresholded so as to only show electrode contacts).

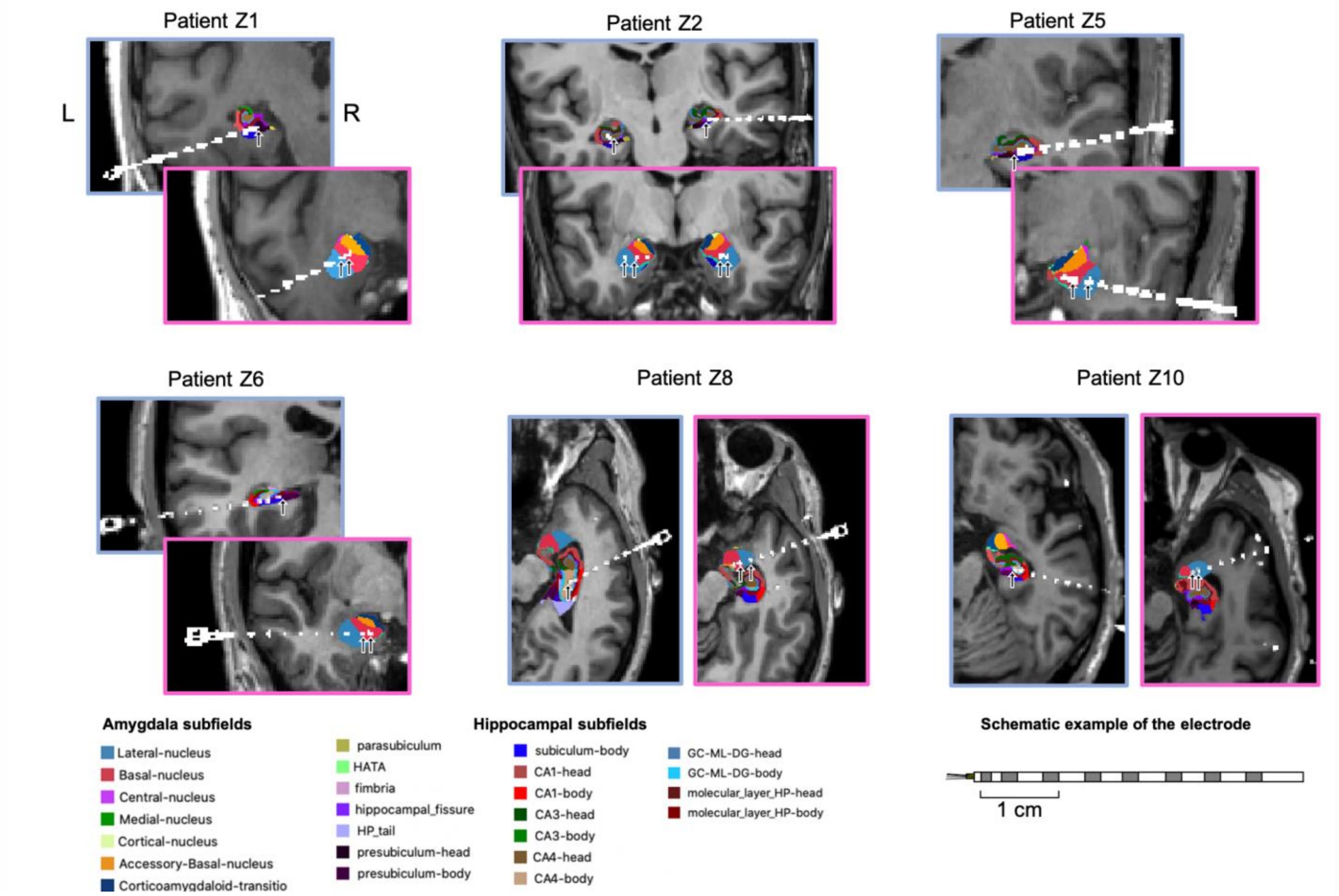

Supplementary Fig. 2: Electrode contact localization for patients (Cohort 2) with macro and micro electrodes localized in the amygdala (pink outline) and hippocampal (light blue outline) subfields. Arrows in the figure representing hippocampal subfields point to the putative microelectrodes location which extends from the first contact as can be seen in the schematic example of the electrode. Arrows in the figure representing the amygdala subfields point to the contacts from where the LFP was extracted for the spike field coherence analysis. For all patients, post-operative CT images from each patient were co-registered with their corresponding pre-operative MRI scans in native space using lead-DBS v2.5 and superimposed to display amygdala and hippocampal contacts (CTs were thresholded so as to only show electrode contacts). Amygdala and hippocampal subfields were obtained using FreeSurfer V.7.2.0. For each subject and brain structure the legend reports the amygdala and hippocampal subfields where contacts are localized. All amygdala and hippocampal subfield colour codes are shown below, and a schematic example of the implanted electrode type (1.3 mm diameter, 8 contacts of 1.6 mm length, and spacing between contact centers 5 mm; Ad-Tech, Racine, WI) is provided.

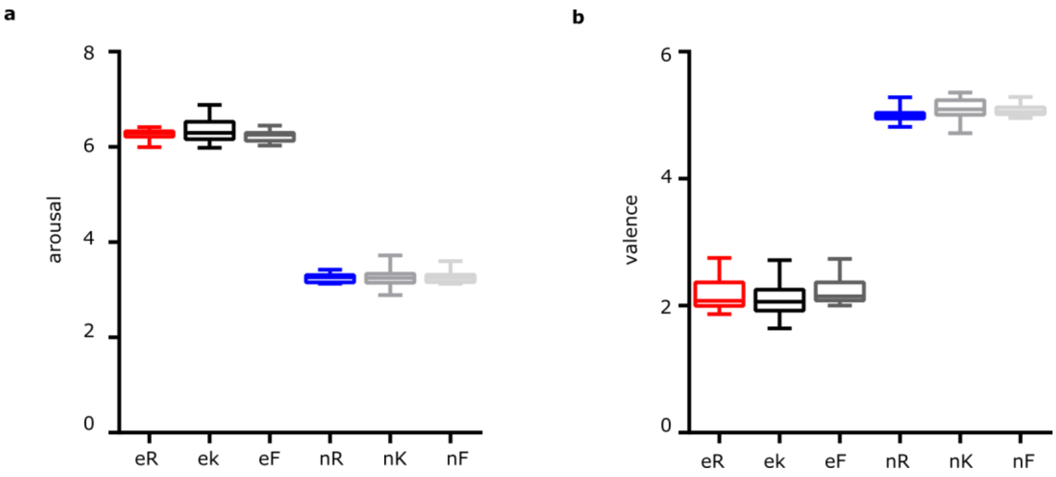

Supplementary Fig. 3. Emotional memory performance: subsequent remembering *vs.* knowing *vs.* forgetting is not related to differences in arousal and valence ratings. Aversive and neutral IAPS pictures were selected on the basis of normative ratings for arousal and valence. To test whether amygdala and hippocampal subsequent memory-dependent spectral responses to emotional scenes could have been driven by different arousal or valence, we performed the following comparisons. **a**, For all stimuli presented at encoding, normative arousal ratings were entered into a repeated measures ANOVA with factors emotion (aversive e, neutral n) and subsequent memory (R, K, F) for both patient cohorts 1 and 2 (n=18 patients). We did not observe a significant emotion by memory interaction ($F_{(2,16)}$=1.93, P=0.169, η^2=0.199). **b**, Repeating this comparison with valence ratings, we again do not show a significant interaction, ($F_{(2,16)}$=2.76, P=0.093, η^2=0.25), (n=18 patients). There was no main effect of subsequent memory for either arousal ($F_{(2,\ 16)}$=0.906, P=0.424, η^2=0.102) or valence rating ($F_{(2,\ 16)}$=3.47, P=0.056, η^2=0.303). The main effect of emotion is significant for both rating parameters, as stimuli were selected on this basis. Bars indicate mean / ± s.e.m. Source Data are provided as a Source Data file.

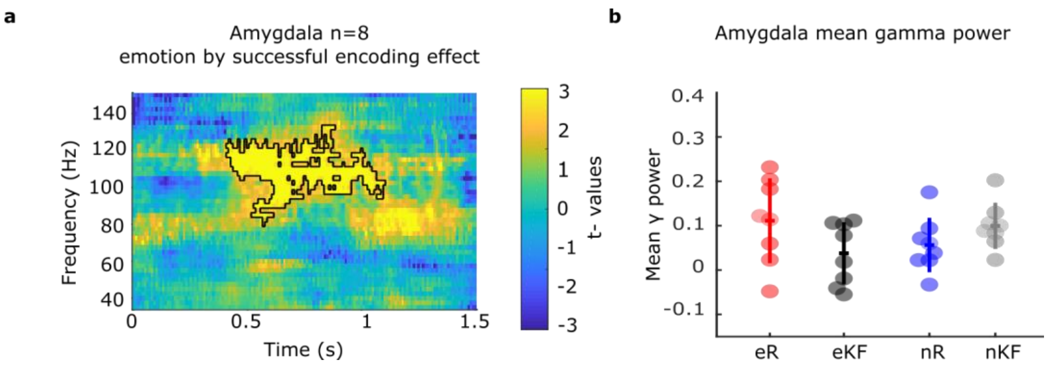

Supplementary Fig. 4. Amygdala gamma power during successful emotional memory formation in the group of patients with electrodes in both the amygdala and ipsilateral hippocampus (Cohort 1). a, Time frequency resolved test statistics for the emotion by subsequent memory effect, (emotion by subsequent memory interaction, summed *t*-value=1898.01, *P*=0.0042), two -sided paired t-test (cluster-based permutation test). Amygdala power change in the high gamma range is greater for eR *vs*. eKF relative to nR *vs*. nKF scenes. The colorbar represents summed *t*-values (post-hoc t-tests on mean power changes show eR *vs.* eKF $t_{7=}$2.40, *P*=0.047, d=0.85; nR *vs.* nKF, $t_{7=}$-2.94, *P*=0.021, d=-1.04). **b,** Mean amygdala gamma power in the significant cluster (from 0.41–1.1 s and from 80–132 Hz) for the four trials types. Circles show individual electrode data, (*n*= 7 patients and 8 electrodes, one patient (Patient Z1) was excluded from power interaction testing as only 2 trials in the neutral remembered condition were obtained). Horizontal / vertical lines indicate mean / ± s.e.m. Source Data are provided as a Source Data file.

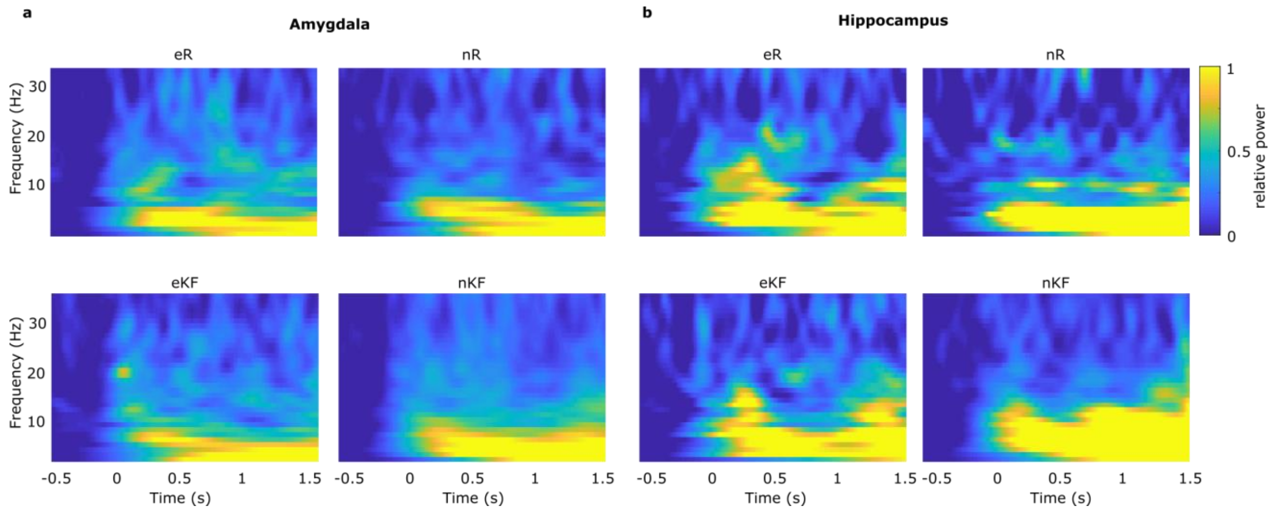

Supplementary Fig. 5. Stimulus-induced low frequency oscillatory power during encoding in amygdala and hippocampus (Cohort 1). Low frequency oscillatory relative power increases, primarily in the delta and theta ranges, are observed to all conditions in both **a**, amygdala and **b,** hippocampus. In neither structure did we observe an emotion by subsequent memory interaction, or main effect. eR: subsequently remembered and eKF know/forgotten aversive pictures; nR subsequently neutral remembered and nKF known/forgotten neutral trials. The colorbar indicates power change relative to baseline.

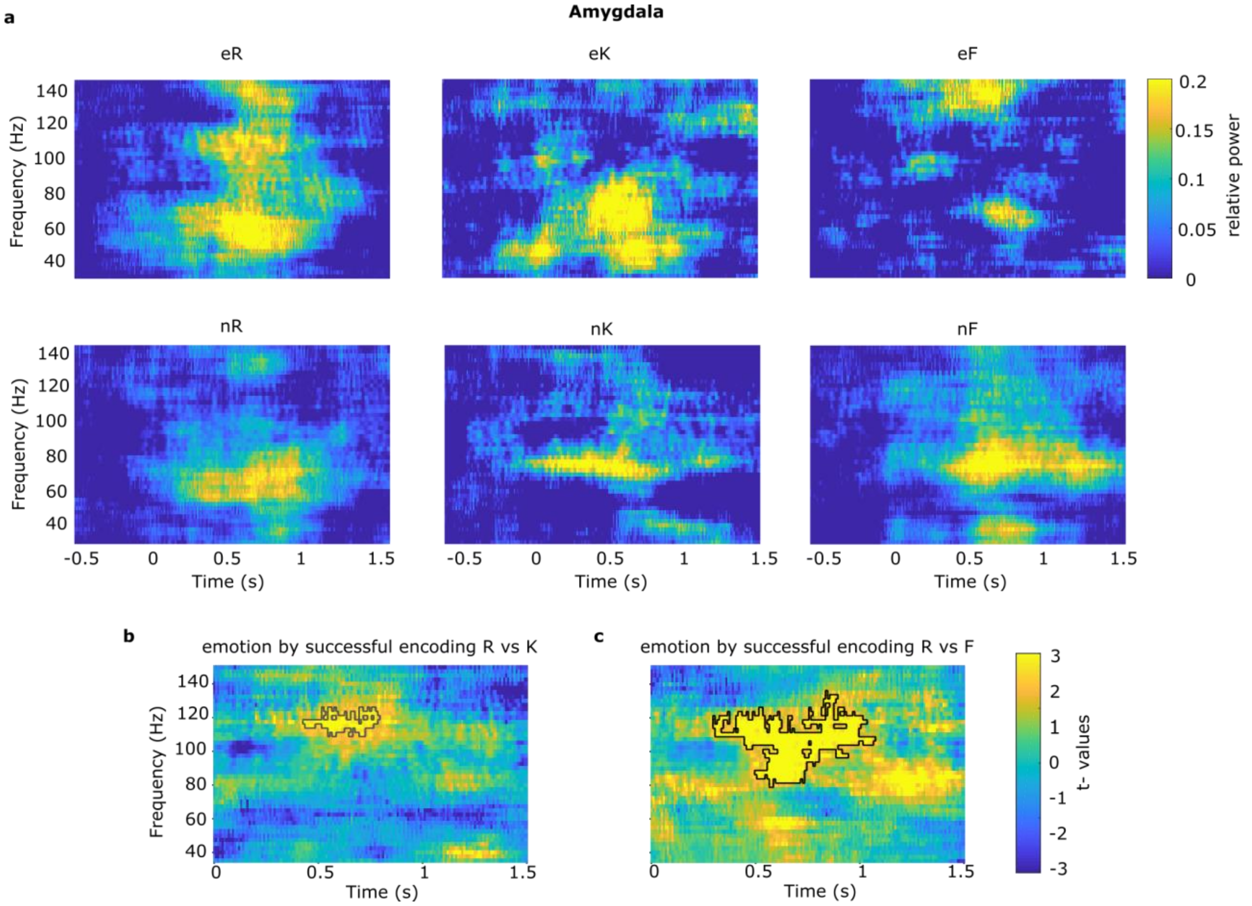

Supplementary Fig. 6. Fast amygdala gamma power during successful emotional memory formation: overlapping frequency effects when comparing gamma responses during aversive subsequent remember *vs.* both known (K) and forgotten (F) trials (Cohort 1). a, Amygdala power change in the gamma range for the six trial types: emotional remember (eR), known (eK), forgotten (eF); neutral remember (nR), known (nK), forgotten (nF). Colorbar depicts relative change from baseline. **b, c** Time frequency resolved test statistics, two-sided paired t-test (cluster-based permutation test), showing overlapping frequency effects when **b,** testing the interaction (eR-eK) *vs*. (nR-nK) (summed *t*-value=438.52, P=0.11, cluster from 0.43–0.79 s and from 110-125 Hz) (n=7 patients; 8 electrodes) and **c,** the interaction (eR-eF) *vs*. (nR-nF) (summed t-value=2306.6, P=0.01, cluster from 0.30–1.07 s and from 80-135 Hz), (n=7 patients; 8 electrodes). No significant gamma power effects ($P > 0.05$) were observed on comparing eK *vs*. eF trials. Colorbar depicts the t-statistic.

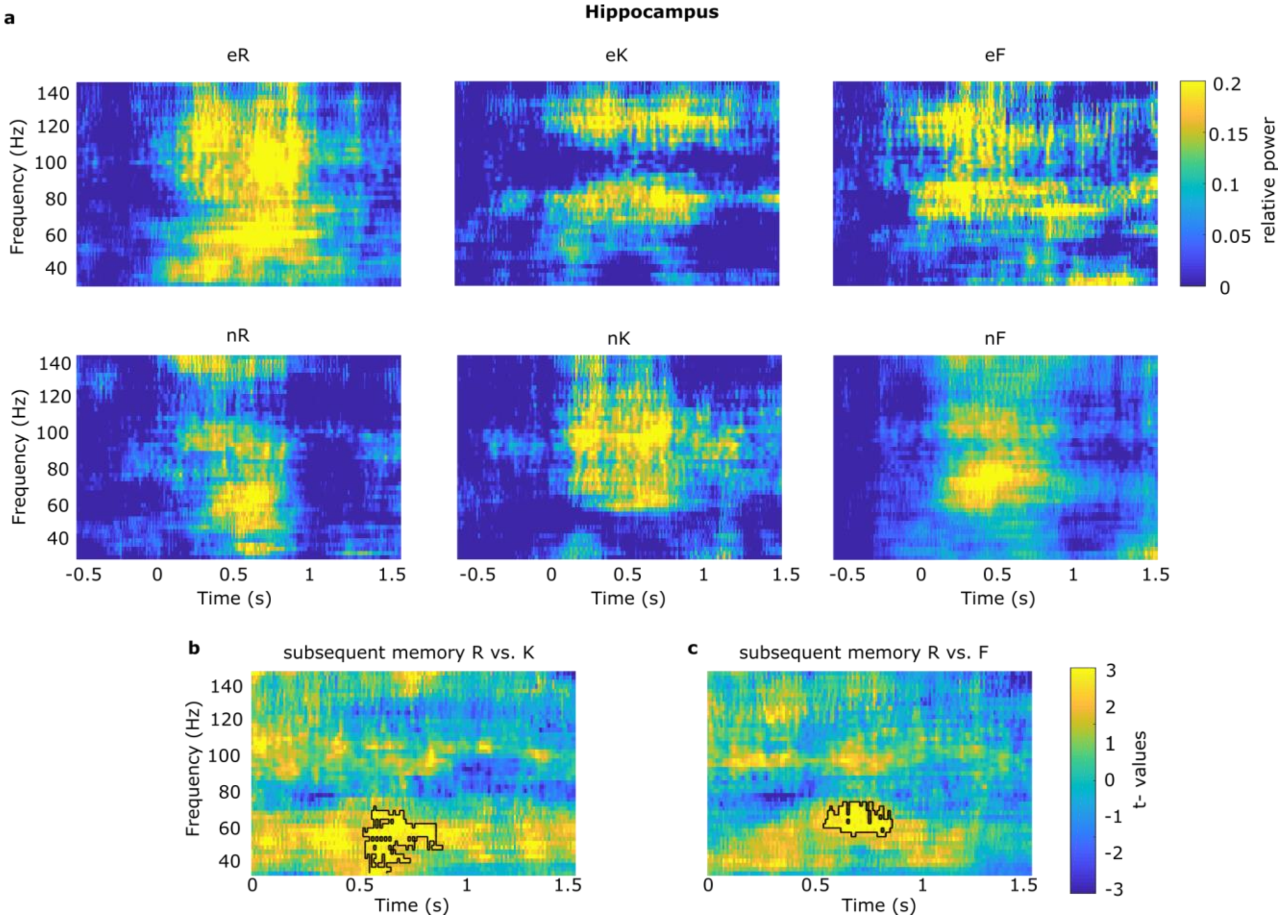

Supplementary Fig. 7. Gamma power in the hippocampus during memory formation: overlapping frequency effects when comparing gamma responses during subsequently remembered *vs.* both known (K) and forgotten (F) trials (Cohort 1). a, Hippocampus power change in the gamma range for the six trial types: aversive remember (eR), known (eK), forgotten (eF); neutral remember (nR), known (nK), forgotten (nF). Colorbar depicts relative change from baseline. **b, c,** Time frequency resolved test statistics, two-sided paired t-test (cluster-based permutation test), showing overlapping frequency effects when comparing gamma responses during **b,** subsequently remembered minus subsequently known (R-K) (summed *t*-value value=778.15, *P*=0.016, cluster from 0.52– 0.88 s and from 35-72 Hz) and **c,** subsequently remembered minus subsequently forgotten (R-F) trials (summed *t*-value=521.47, *P*=0.024, cluster from 0.54– 0.85 s and from 57-75 Hz), (*n*=8 patients; 9 electrodes). No significant gamma power effects (*P* > 0.05) were observed on comparing K *vs*. F trials in the hippocampus. Colorbar depicts the *t*-statistic.

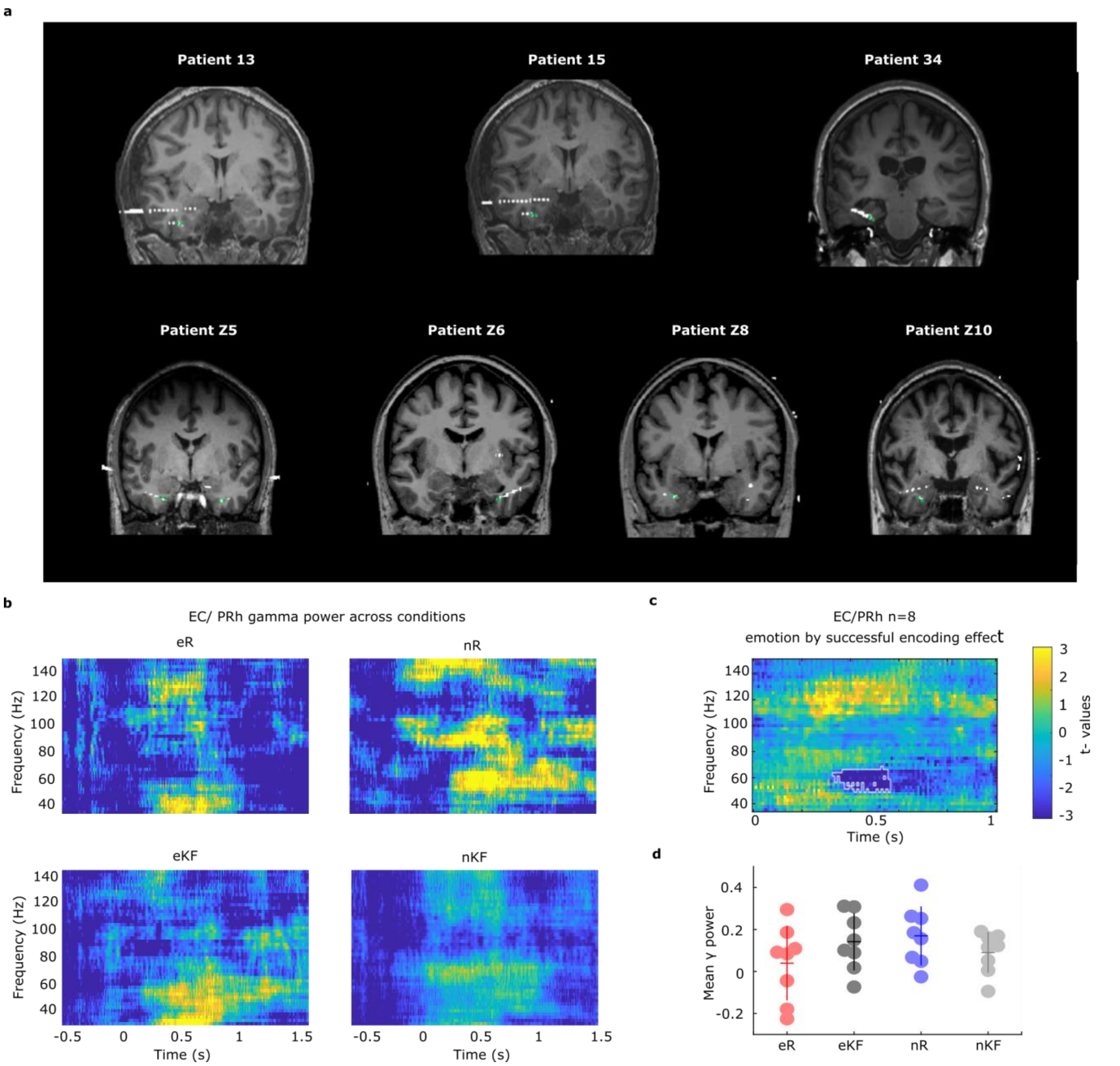

Supplementary Fig. 8. Entorhinal (EC) and Perirhinal (PRh) cortex gamma activity during memory formation of neutral, but not emotional, scenes. EC/PRh recordings were pooled over Cohorts 1 and 2. **a,** Electrode contact localization for the 7 patients with macro-electrodes localized in the Entorhinal (EC) or Perirhinal (PRh) cortex. Green dots indicate contacts included in the analyses. **b,** EC/PRh power change in the gamma range for the four trial types: emotional remember (eR), known/forgotten (eKF); neutral remember (nR), known/forgotten (nKF). Colorbar depicts relative change from baseline. **c**, Time frequency resolved test statistics, two-sided paired t-test (cluster-based permutation test), for the emotion by subsequent memory effect. EC/PRh power change in the gamma range is greater for nR *vs.* nKF relative to eR *vs.* eKF scenes, (emotion by subsequent memory interaction, summed *t*-value=-475.27, *P*=0.023). The colorbar indicates *t*-values. (eR *vs.* eKF t_7=-2.94, *P*=0.021; nR *vs.* nKF t_7= 3.10, *P*=0.017). **d**, Mean gamma power in the significant cluster (from 0.33 –0.56 s and from 50–67 Hz) for the four trials types. Circles show individual electrode data (*n*=7 patients, 8 electrodes). Horizontal / vertical lines indicate mean / ± s.e.m. Source Data are provided as a Source Data file.

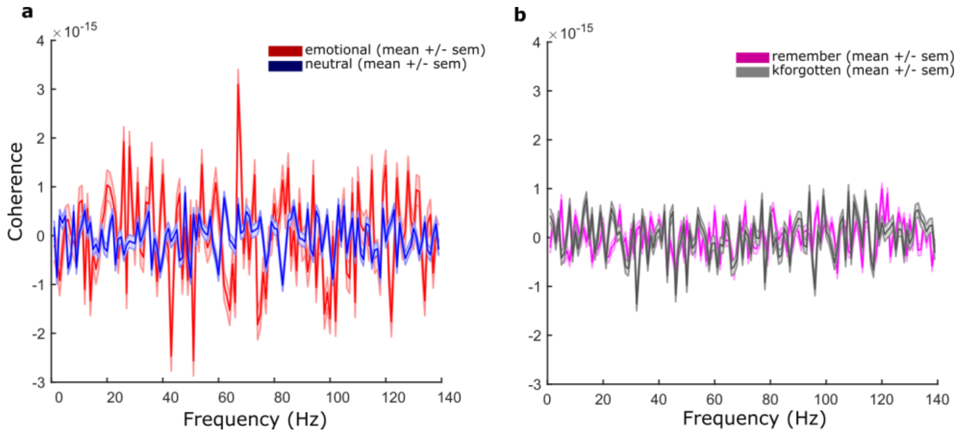

Supplementary Fig. 9. Coherence between stimulus-induced amygdala and hippocampal activity. Frequency-resolved coherence (mean ± standard error) between amygdala and hippocampus (Cohort 1, *n*= 8 patients; 9 amygdala-hippocampal electrode pairs) is plotted during **a,** emotional *vs*. neutral trials, and **b,** remember *vs*. known and forgotten trials. No main effects in any frequency were found for either comparison, nor did coherence values show an emotion (aversive, neutral) by subsequent memory (R, KF) interaction.

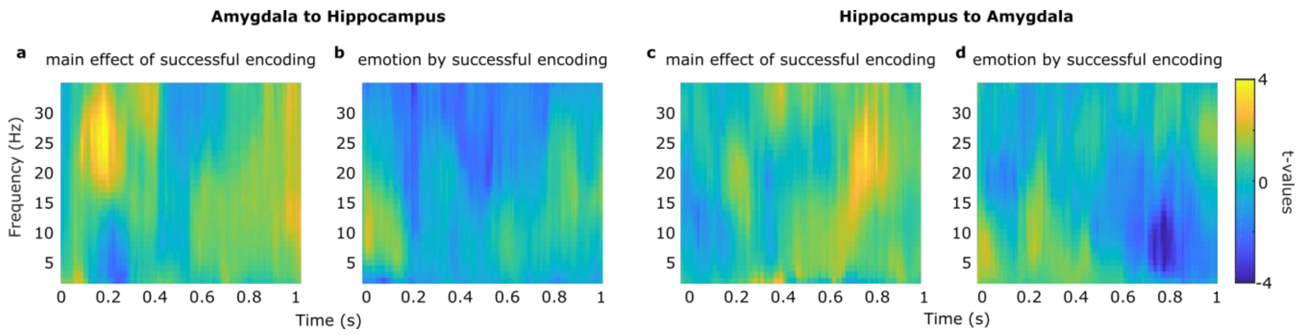

Supplementary Fig. 10. Time and frequency-resolved Granger causality (GC) between amygdala and hippocampal activity (Cohort 1). Time and frequency resolved GC in the amygdala to hippocampus direction is plotted for **a**, the main effect of successful encoding (Remember vs Known/Forgotten and **b**, the interaction emotion by successful encoding (eR-eKF) *vs.* (nR - nKF). The same measures, but in the hippocampus to amygdala direction, are plotted for **c**, the main effect of successful encoding and **d**, the interaction emotion by successful encoding. No effect survived cluster-based correction.

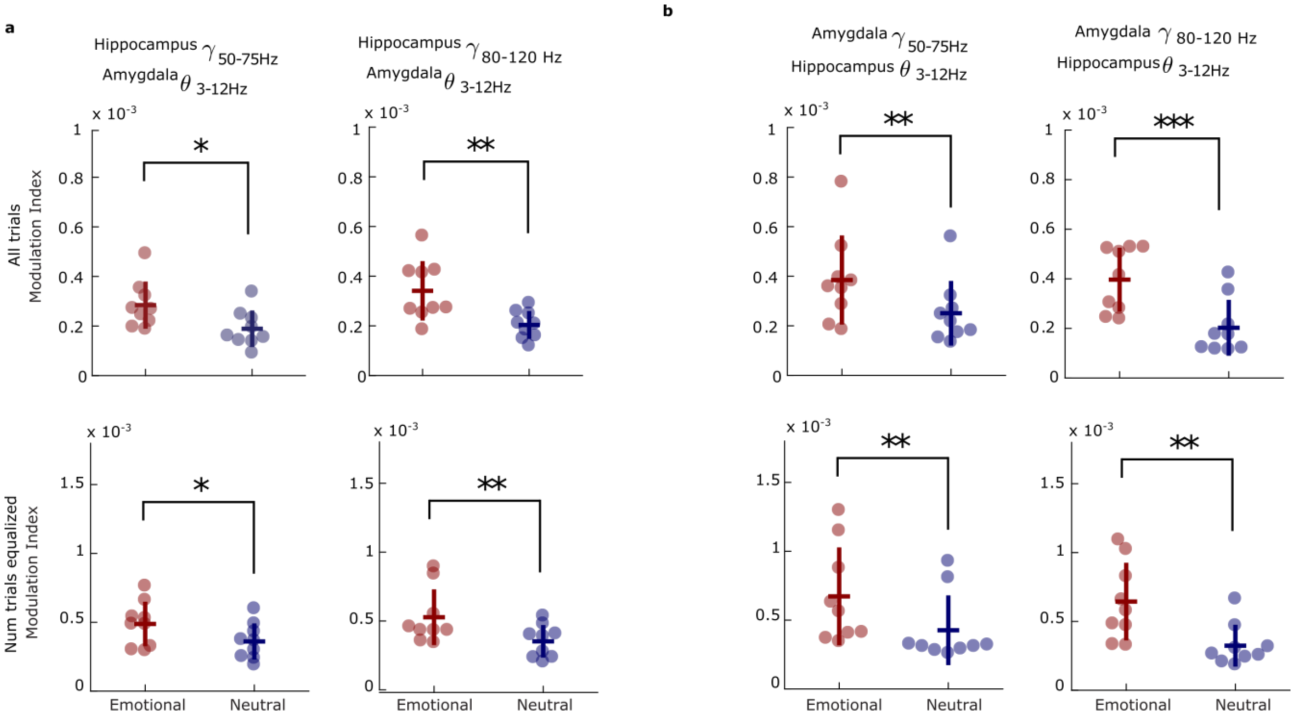

Supplementary Fig. 11. **Between-region theta-gamma phase-to-amplitude coupling (Cohort 1).** **a**, Modulation index for PAC between amygdala theta phase frequency (3–12 Hz) and hippocampus gamma amplitude, in the 50–75 Hz range (upper left, identical to Fig. 3d in the main text) and hippocampus gamma amplitude in the 80–120 Hz range (main effect of emotion: $F_{(1, 8)}$=22.9, P=0.0014, η^2=0.741), * P < 0.05; ** P < 0.005; *** P< 0.0005, repeated measure ANOVA. Lower left and right panels show the same conditions but controlling for the number of trials between conditions ($F_{(1, 8)}$=6.65, P=0.033, η^2=0.454; $F_{(1, 8)}$=12.6, P=0.0075, η^2=0.612, respectively; see Materials and Methods). For each dot plot, the modulation index for the emotion condition (eR+eKF) is shown in red and the neutral condition (nR+nKF) in blue, (n=8 patients; 9 amygdala-hippocampal electrode pairs). Horizontal and vertical bars represent the mean and s.e.m., respectively. Each dot represents a single bipolar channel in a given experimental condition. **b**, Modulation index for PAC between hippocampus theta phase frequency (3–12 Hz) and amygdala gamma amplitude, with same figure conventions as in **a**. Upper left, hippocampus theta phase to amygdala gamma 50–75 Hz amplitude (main effect of emotion: $F_{(1, 8)}$=20.3, P=0.0020, η^2=0.718); and upper right, amygdala gamma 80-120 Hz amplitude (main effect of emotion: $F_{(1, 8)}$=44.2, P=0.00016, η^2=0.847). Lower left and right show the emotion main effect controlling the number of trials between conditions (amygdala gamma 50–75 Hz amplitude, $F_{(1, 8)}$=15.1, P=0.0046, η^2=0.654; amygdala gamma 80–120 Hz amplitude, $F_{(1, 8)}$=19.7, P=0.0022, η^2=0.711). All reported main effects were computed including lateral bipolar channels from all patients with simultaneous hippocampus and amygdala recordings (n=8 patients, 9 amygdala-hippocampal electrode pairs) during 0.41–1.1 s post-stimulus onset.

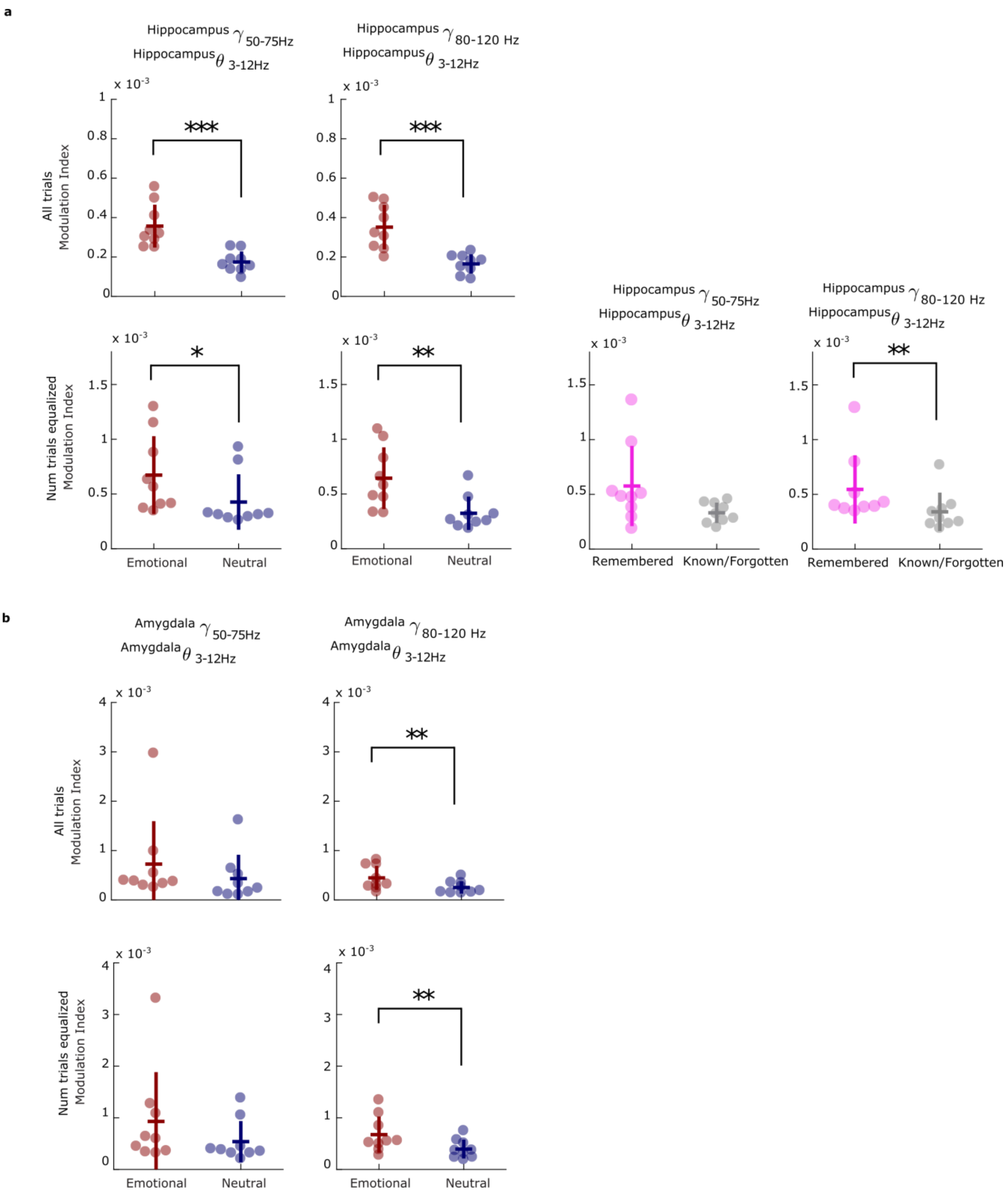

Supplementary Fig. 12. **Within-region theta-gamma phase-to-amplitude coupling (Cohort 1). a**, Modulation index for PAC between hippocampus theta phase frequency (3–12 Hz) to hippocampus gamma amplitude, * P < 0.05; ** P < 0.005; *** P< 0.0005, repeated measure ANOVA. Upper left: hippocampus gamma amplitude 50–75 Hz; emotion main effect $F_{(1, 8)}$=27.5, *P*=0.00078, η^2=0.775; Upper right: hippocampus gamma amplitude 80–120 Hz; emotion main effect $F_{(1, 8)}$=42.8, *P*=0.00018, η^2=0.843. Lower two left panels show the same conditions but controlling for the number of trials between conditions ($F_{(1, 8)}$=10, *P*=0.013, η^2=0.556; $F_{(1, 8)}$=16.6, *P*=0.0036, η^2=0.675, respectively). For each dot plot, the modulation index for the emotion condition (eR+eKF) is shown in red and the neutral condition (nR+nKF) in blue (*n* =8 patients, 9 amygdala-hippocampal electrode pairs). Horizontal and vertical bars represent the mean and s.e.m.,

respectively. Each dot represents a single bipolar channel in a given experimental condition. The bottom right two dot plots show the modulation index for hippocampal theta phase to hippocampal gamma as a function of subsequent memory, with number of trials equalized between subsequently remembered (eR+nR, magenta) and not remembered (eKF+nKF, grey) trials. The main effect of memory was not significant for the hippocampus 50–75 Hz gamma band ($F_{(1, 8)}$=5.08, P=0.054, η^2=0.389) but it was evident for the hippocampus 80–120 Hz band ($F_{(1, 8)}$=16.6, P=0.0036, η^2=0. 675). **b**, Modulation index for PAC between amygdala theta phase frequency (3–12 Hz) to amygdala gamma amplitude, repeated measure ANOVA. Upper left: amygdala gamma amplitude 50–75 Hz; emotion main effect $F_{(1, 8)}$=4.07, P=0.08, η^2=0.337; Upper right: hippocampus gamma amplitude 80–120 Hz; emotion main effect $F_{(1, 8)}$=14.4, P=0.0052, η^2=0.664. Lower two left panels show the same conditions but controlling for the number of trials between conditions ($F_{(1,8)}$=2.69, P=0.14, η^2=0.252; $F_{(1,8)}$=12.2, P=0.0082, η^2=0.603, respectively). All reported main effects were computed including lateral bipolar channels from all patients with simultaneous hippocampus and amygdala recordings (*n* =8 patients, 9 amygdala-hippocampal electrode pairs) during 0.41–1.1 s post-stimulus onset.

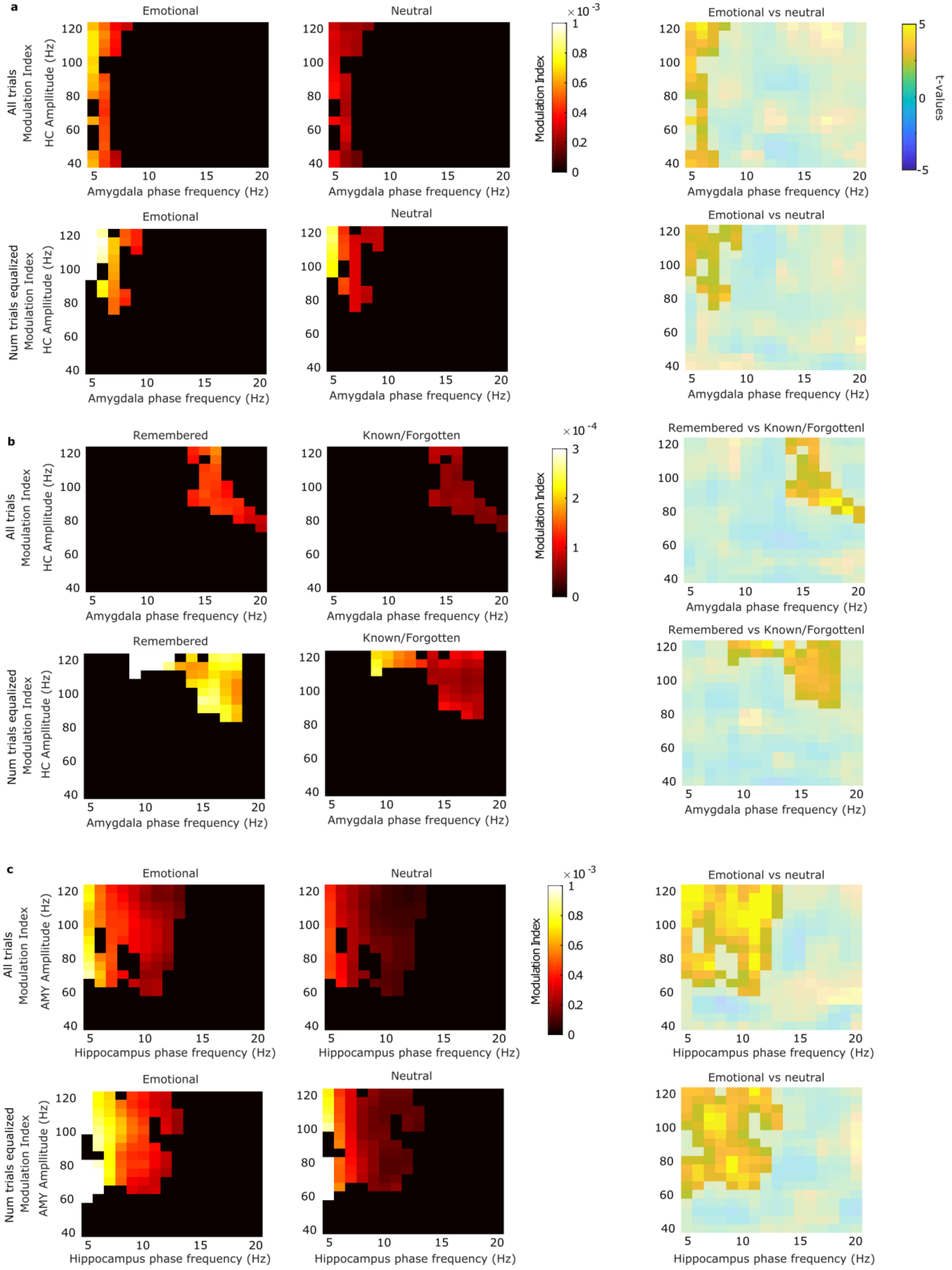

Supplementary Fig. 13. Between-region phase-to-amplitude coupling comodulograms (Cohort 1). **a**, Amygdala phase frequency (x-axes) to hippocampus amplitude (y-axes) coupling during emotional (top left) and neutral (top middle) conditions. Color code represents the modulation index (see Materials and Methods). Group-level statistics map (top right) ensuing from the main effect of emotion (eR+eKF *vs.* nR+nKF) contrast where color code represents the *t*-score (summed *t*-value=104.95, *P*=9.99 x 10^{-5}), two-sided paired t-test

(cluster-based permutation test). In this analysis all trials for each condition were included. Bottom row uses the same convention as top row but controlling the number of trials per condition (summed *t*-value=73.70, *P*=0.0116). **b**, Same conventions as in **a** but representing the main effect of memory (eR+nR *vs.* eKF+nKF) for amygdala phase to hippocampus amplitude (all trials, summed *t*-values=89.87, *P*=0.017; number of trials equalized summed *t*-value=127.51, *P*=0.0063). **c**, Same figure conventions as **a** and **b** but showing the main effect of emotion for hippocampus phase to amygdala amplitude coupling (all trials, summed *t*-value=347.55, P=9.99 x 10^{-5}, number of trials equalized, summed *t*-values=279.02, P=9.99 x 10^{-5}, respectively). All reported main effects were computed including lateral bipolar channels from all patients with simultaneous hippocampus and amygdala recordings (*n*=8 patients, 9 amygdala-hippocampal electrode pairs) during 0.41–1.1 s post-stimulus onset.

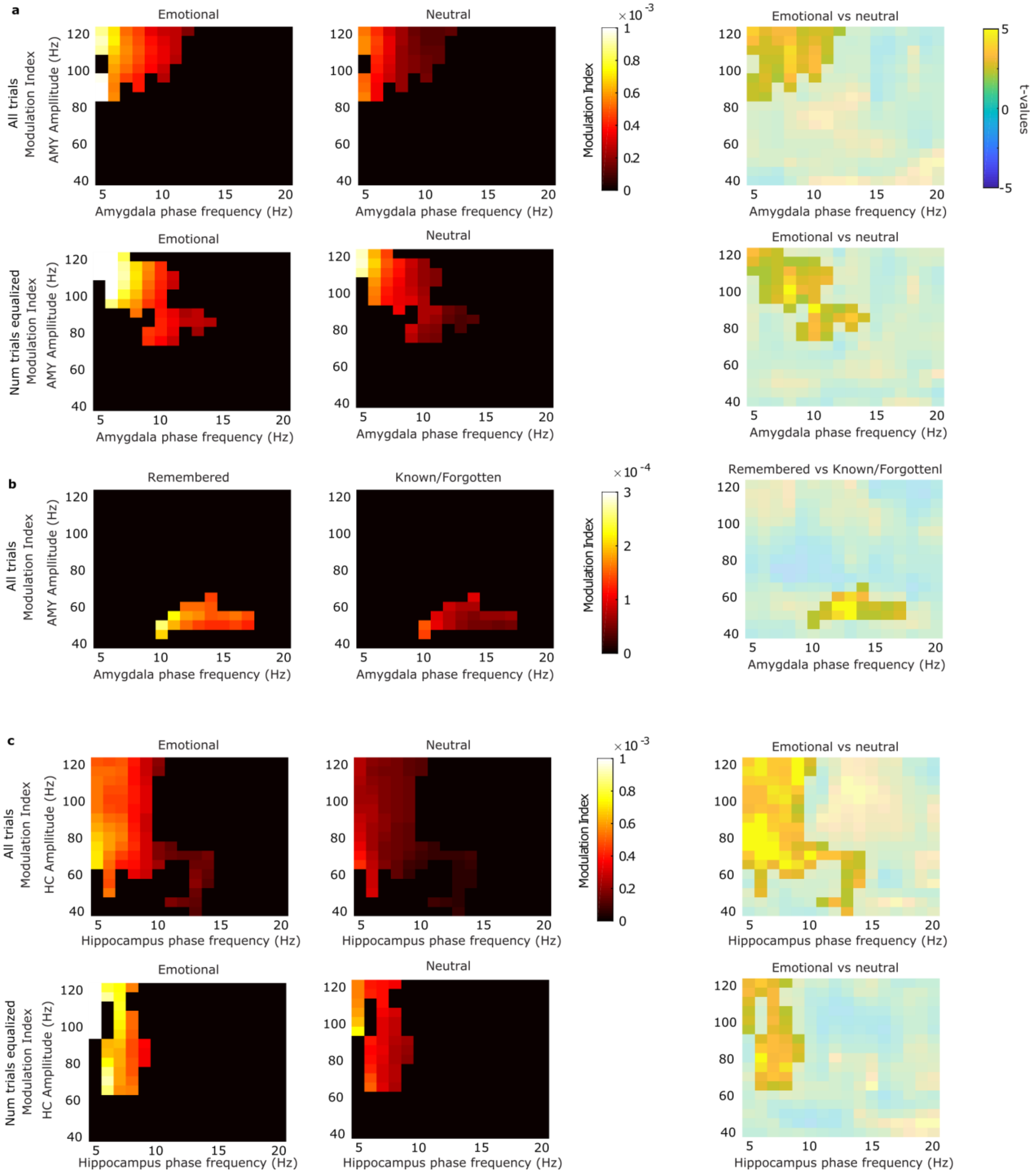

Supplementary Fig. 14. **Within-region phase-to-amplitude coupling comodulograms (Cohort 1). a**, Amygdala phase frequency (x-axes) to amygdala amplitude (y-axes) coupling during emotional (top left) and neutral (top middle) conditions. Color code represents the modulation index. Group-level statistics map (top right) ensuing from the main effect of emotion (eR+eKF vs. nR+nKF) contrast where color code represents the T-score (summed *t*-value=121.44, *P*=0.0047), two-sided paired t-test (cluster-based permutation test). In this analysis all trials for each condition were included. Bottom row uses the same convention as top row but controlling the number of trials per condition (summed *t*-value=136.83, *P*=9.99 x 10^{-5}). **b**, Same conventions as in **a** but representing the main effect of memory (eR+nR *vs*. eKF+nKF) for amygdala phase to amygdala amplitude coupling (all trials, summed *t*-value=63.13, *P*=0.024; not significant when number of trials were equalized). **c**, Same figure conventions as in **a** but showing the main effect of emotion for hippocampus phase to hippocampus amplitude coupling (summed *t*-value=329.19, *P*=9.99 x 10^{-5}, summed *t*-value=119.05, *P*=9.99 x 10^{-5}, respectively). All reported main effects were computed including lateral bipolar channels from all patients with simultaneous hippocampus and amygdala recordings (*n*=8 patients, 9 amygdala-hippocampal electrode pairs) during 0.41–1.1 s post-stimulus onset.

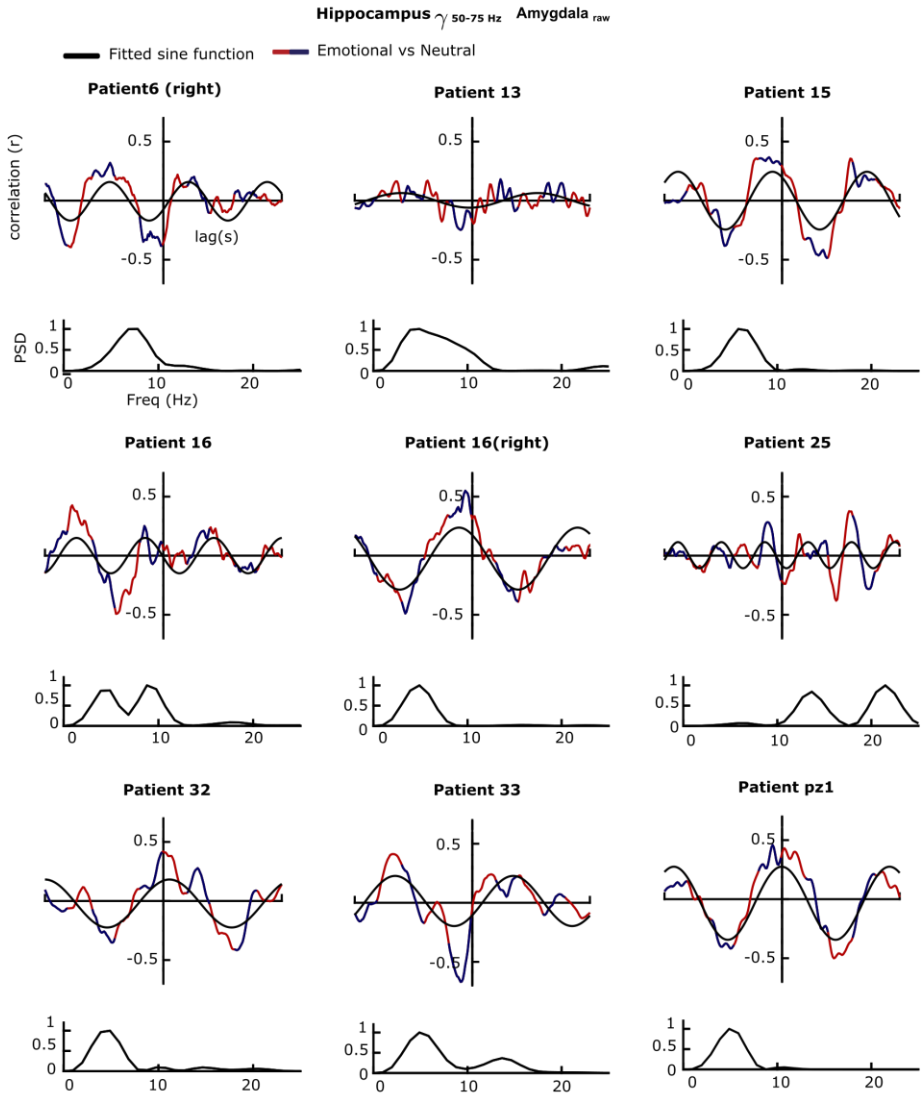

Supplementary Fig. 15. Inter-regional cross-correlation between emotional *vs.* neutral peak triggered average (PTA; blue/red) for each patient's amygdala-hippocampus pair (Cohort 1). Each PTA was computed by first filtering hippocampus gamma activity (50–75 Hz), then identifying gamma peaks (minimum separation 0.1 s) and finally averaging the raw traces from amygdala recordings (± 0.12 s) centered (t=0 s) around the hippocampus gamma peaks (see Materials and Methods). Each subplot represents a lateral amygdala and lateral hippocampus bipolar channel pair. Bottom insert: power spectral density (PSD) taken over the entire cross-correlogram displays the main spectral component that dominates the PTA. The PSD peak was used to fit the optimal sine wave (black), in the least squares sense. Note that all amygdala traces show a theta component reflected in the PSD and in the fitted (black) sine wave. All reported main effects were computed including lateral bipolar channels from all patients with simultaneous hippocampus and amygdala recordings (n=8 patients, 9 amygdala-hippocampal electrode pairs) during 0.41–1.1 s post-stimulus onset.

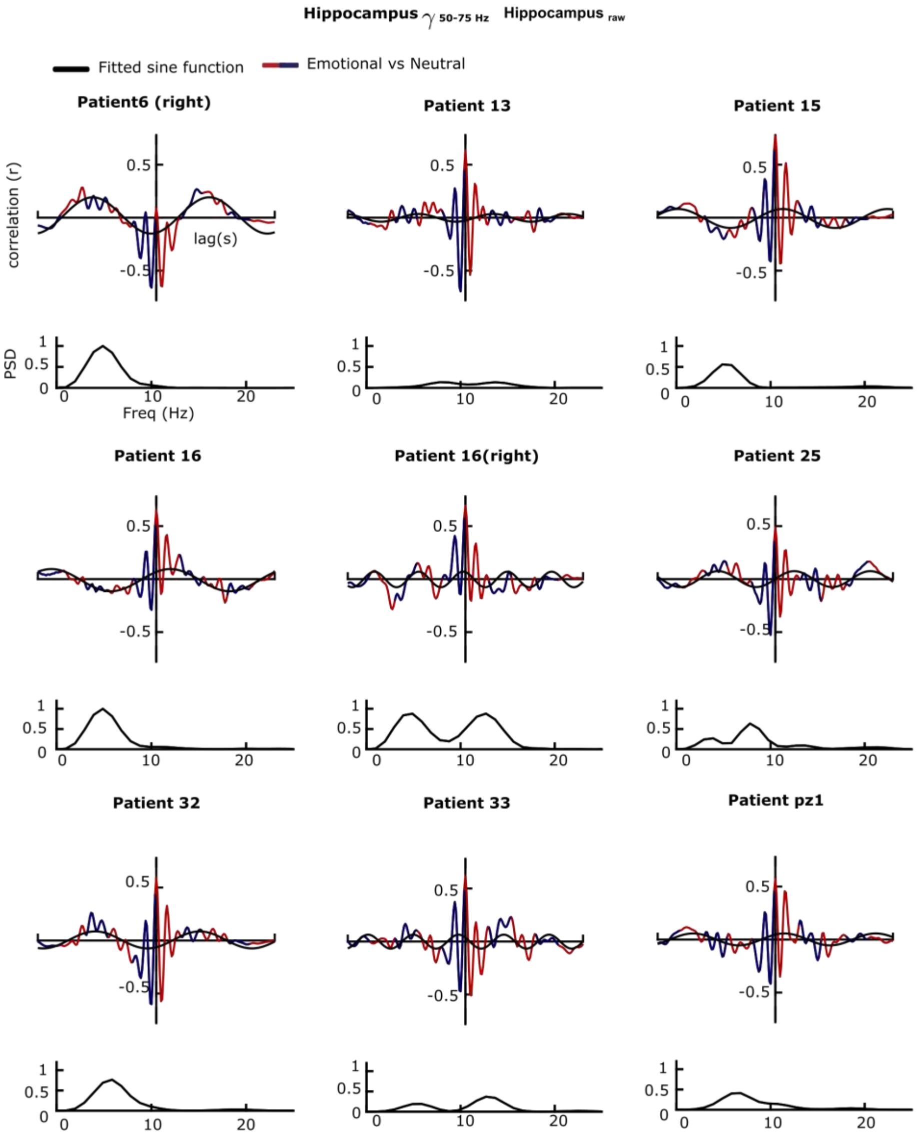

Supplementary Fig. 16. Single contact within-hippocampus cross-correlation between emotional *vs.* neutral peak triggered average (PTA; dark blue/dark red) (Cohort 1). Each PTA was computed by filtering hippocampus gamma activity (50–75 Hz), then identifying gamma peaks (minimum separation 0.1 s) and finally averaging the raw traces from the same hippocampus recordings (± 0.12 s) centered (t=0 s) around the peaks (see Materials and Methods). Each subplot represents a lateral hippocampus bipolar channel. Bottom insert: power spectral density (PSD) taken over the entire cross-correlogram displays the main spectral component that dominates the PTA. The PSD peak was used to fit the optimal sine wave (black), in the least squares sense. Note that all hippocampal traces show a theta component reflected in the PSD and in the fitted (black) sine wave. All reported main effects were computed including lateral hippocampal bipolar channels from all patients with simultaneous hippocampus and amygdala recordings (n=8 patients, 9 hippocampal electrodes) during 0.41–1.1 s post-stimulus onset.

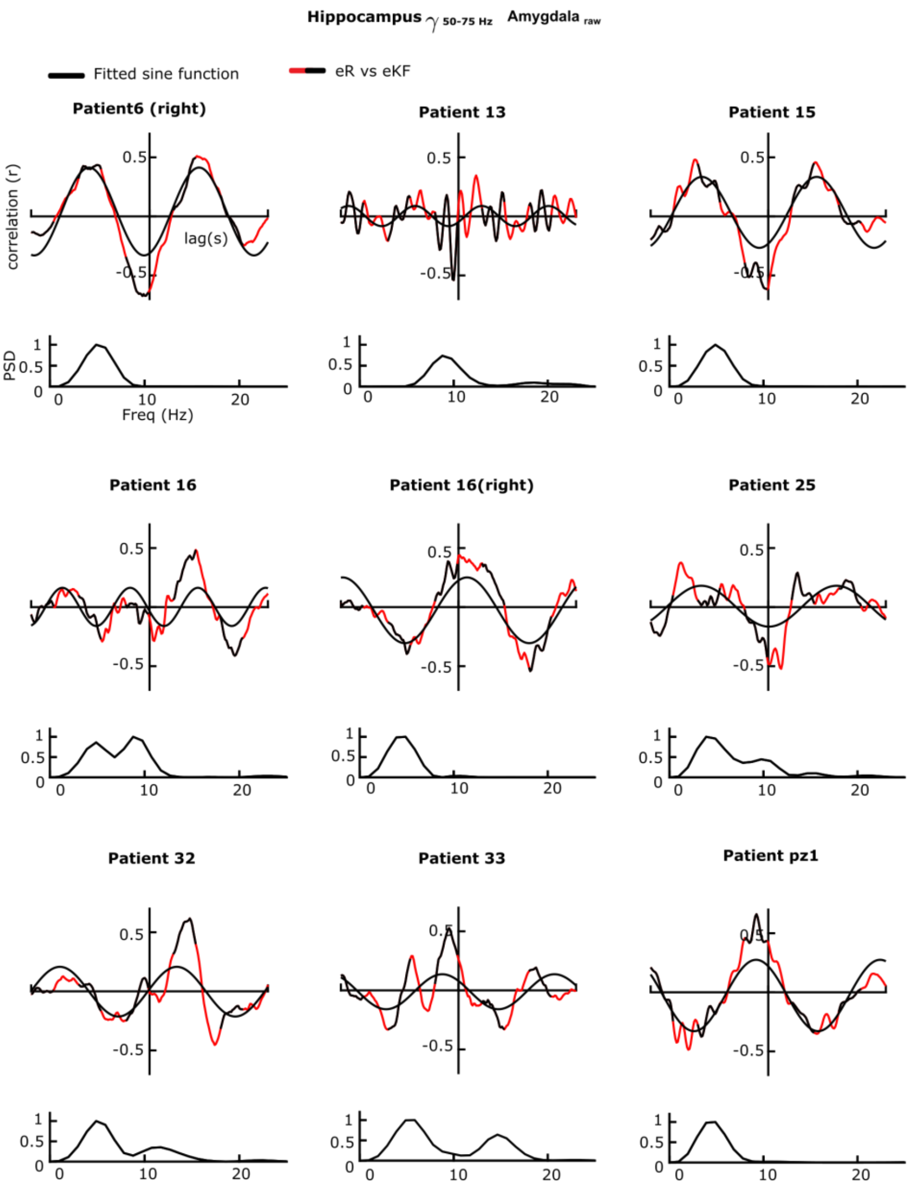

Supplementary Fig. 17. Inter-regional cross-correlation between emotionally remembered (eR) *vs.* emotionally known plus forgotten (eKF) peak triggered average (PTA; red/black) (Cohort 1). Each PTA was computed by filtering hippocampus gamma activity (50 –75 Hz), then identifying gamma peaks (minimum separation 0.1 s) and finally averaging the raw traces from amygdala recordings (± 0.12 s) centered (t=0 s) around the hippocampus gamma peaks (see Materials and Methods). Each subplot represents a lateral amygdala and lateral hippocampus bipolar channel pair. Bottom insert: power spectral density (PSD) taken over the entire cross-correlogram displays the main spectral component that dominates the PTA. The PSD peak was used to fit the optimal sine wave (black), in the least squares sense. Note all amygdala traces show a theta component reflected in the PSD and in the fitted (black) sine wave. All reported simple effects (eR *vs.* eKF) were computed including lateral bipolar channels from all patients with simultaneous hippocampus and amygdala recordings (n=8 patients; 9 amygdala-hippocampal electrode pairs) during 0.41–1.1 s post-stimulus onset.

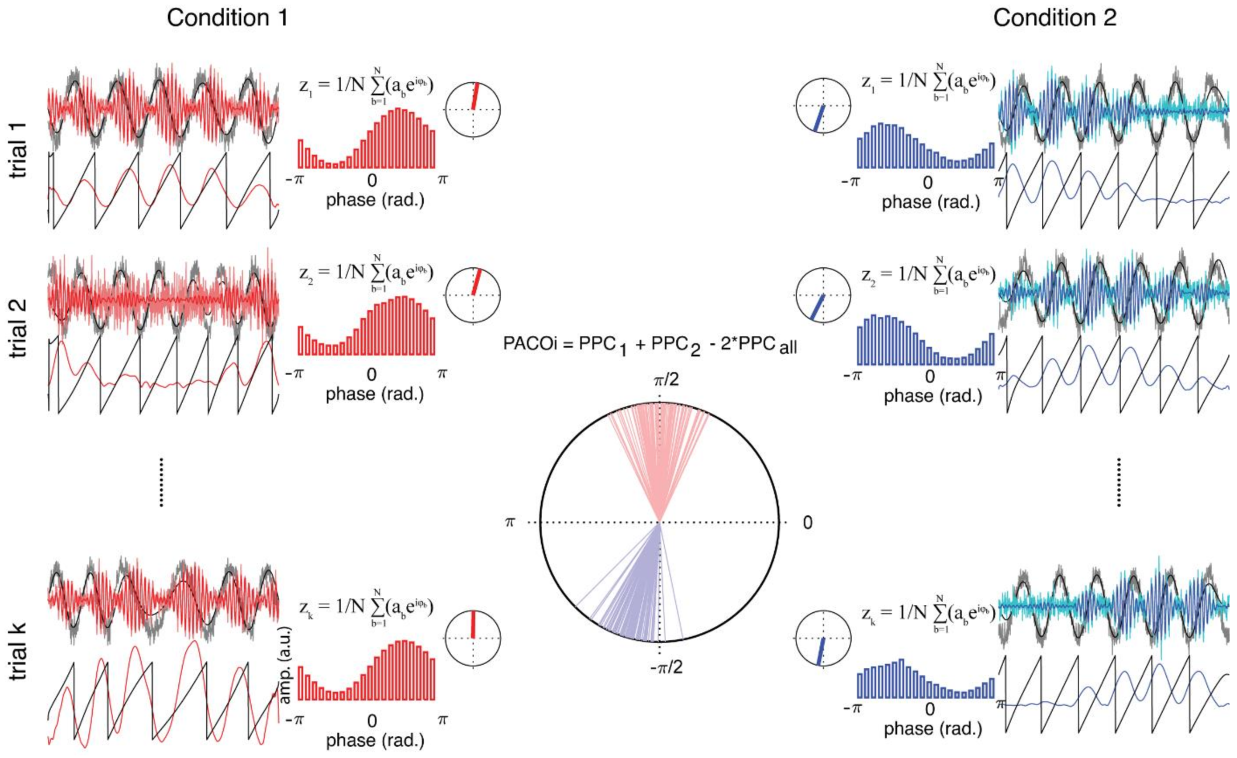

Supplementary Fig. 18. **Phase-Amplitude Coupling Opposition index (PACOi).** PACOi measures whether the high frequency amplitude of condition 1 *vs*. 2 locks at different phase bins. If so, the sum of the pairwise-phase consistency (PPC) of each experimental condition will exceed the PPC obtained from all trials together. This simulation shows the analysis steps for two conditions (left and right columns). The time series are filtered into low (left column; black traces) and high frequencies (left column: red traces) and the analytic phase and amplitude are computed taking the Hilbert transform respectively. The analytic phase of the low frequency is binned (n=20) and the high frequency amplitude is averaged within each phase bin, forming a histogram (left column; red histogram). For each trial, this histogram is transformed into a complex number (z_k; inserted formula) by multiplying the high frequency amplitude (histogram y-axis: ab) by the low frequency phase (histogram x-axis: exp($i^{\varphi b}$); where φ_b denotes the average phase of each bin). This z value is a weighted average that indicates the phase bin of the lower-frequency oscillation at which the amplitude of the high-frequency oscillation is strongest. The z_k complex values were taken for each experimental trial and were normalized to unit length. The right column shows the same for condition 2. Middle column: the phase opposition is based on the sum of the PPC of each condition relative to the PPC calculated taking together the trials of the two conditions ($PACOi=PPC_{eR}+PPC_{eKF}-2*PPC_{eR,eKF}$). The PPC is defined as

$$PPC = \left(\hat{Z} * conj(\hat{Z}) - N\right)/\left(N * (N-1)\right) \quad (1)$$

and

$$\hat{Z} = \sum_{k=1}^{N} Z_k \quad (2)$$

being the sum of single trial complex z values (N=total number of trials). PPC expected value runs from 0 (uniform phase distribution) and 1 (maximum pairwise phase consistency). Negative value may appear due to unbiasedeness of the PPC (see Vinck 2010 for details)[1]. Statistical significance was established by permuting (n=1000 times) the trials of each experimental conditions for a number of pair frequencies (5–20 Hz in 1 Hz steps and 40–120 Hz in 5 Hz steps). For each patient and bipolar channel, we selected the frequency phase-amplitude pairs that show a significant PACOi (uncorrected).

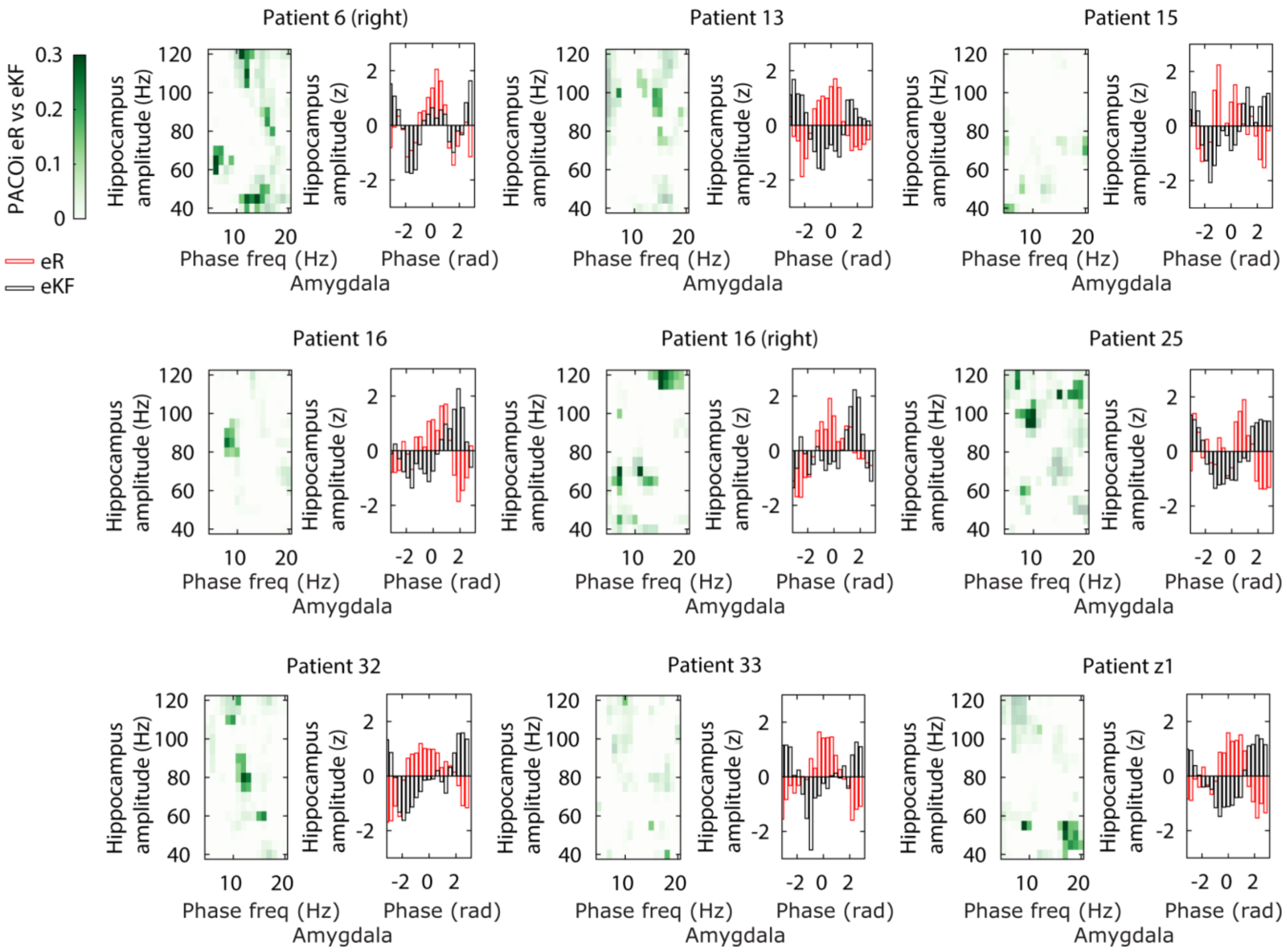

Supplementary Fig. 19. **Phase-Amplitude Coupling Opposition index (PACOi) for aversive remembered and aversive known/forgotten trials for individual amygdala-hippocampal electrode pairs (Cohort 1).** For each pair, left: comodulogram showing the amygdala phase frequency (x-axes) to hippocampus amplitude (y-axes) phase opposition between emotional remembered (eR) *vs*. emotional known + forgotten (eKF) trials. Colorbar indicates the PACOi values masked by (uncorrected) statistical contrast between the eR *vs*. eKF group of trials. For each amygdala-hippocampal electrode pair, right: averaged hippocampus amplitude to amygdala phase histogram resulting from the significant (masked comodulogram) PACOi. Red represents eR and black eKF. PACOi analyses were performed over the 0.41–1.1 s post-stimulus time interval.

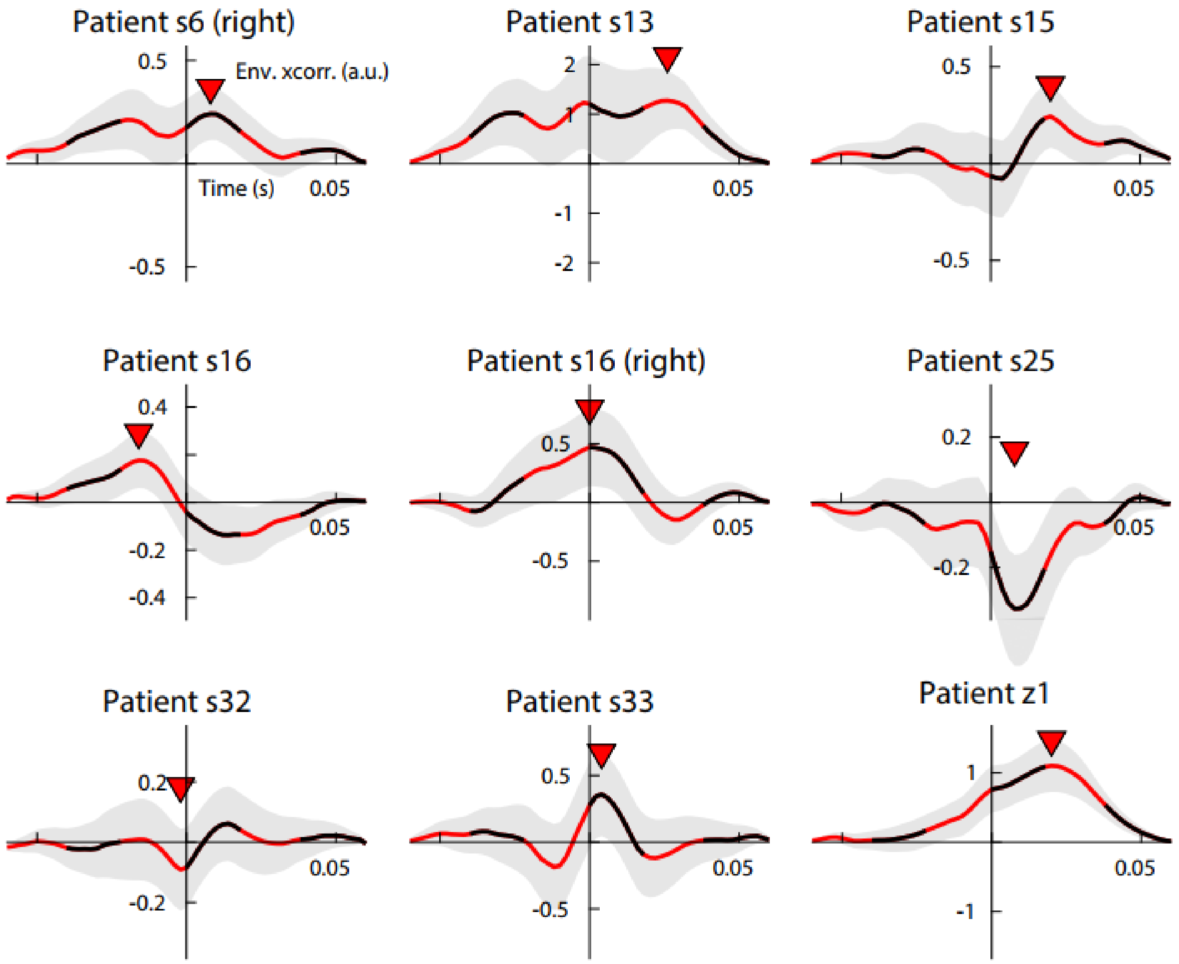

Supplementary Fig. 20. Broadband gamma (60–120 Hz) transient connectivity analysis between hippocampus and amygdala assessed by the amplitude envelope of cross-correlation (Cohort 1). Red/black curves represent the average amplitude envelope cross-correlation (Env. xcorr.) contrast between eR *vs*. eKF. The input signals to compute the cross-correlation were the hippocampus and amygdala broadband gamma activities taken over the 0.41–1.1 s post-stimulus time interval. Red triangles represent the peak lag of the cross-correlation used in Fig 5b. Shaded grey area represents the s.e.m. taken over the epochs, a.u.: arbitrary units.

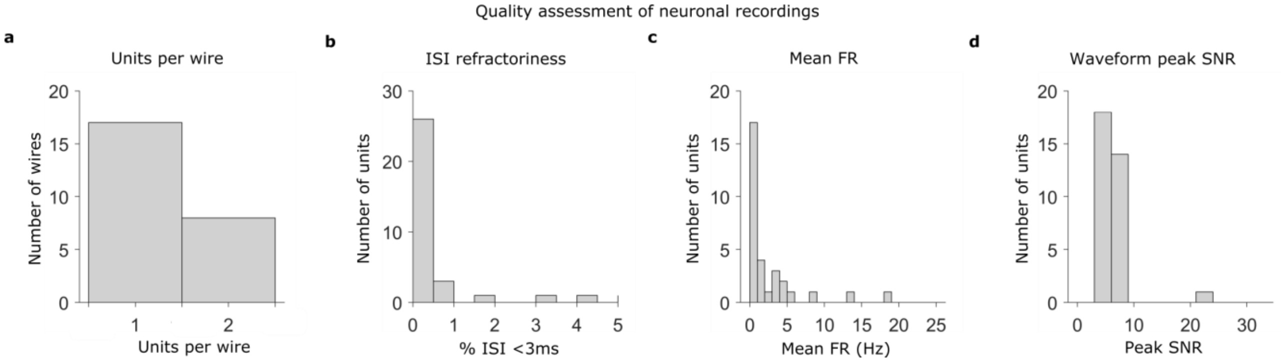

Supplementary Fig. 21. Quality assessment of neuronal recordings and spike sorting (Cohort 2). We recorded *n*=33 neurons from the hippocampus. **a,** Histogram of how many neurons were identified on each wire. On average, 1.32 ± 0.09 neurons per wire (mean ± s.e.m) were recorded. Only wires with at least one neuron are counted. **b,** Histogram of the proportion of inter-spike interval (ISIs) shorter than 3 ms. On average, units exhibited 0.65 ± 0.27% ISIs that were shorter than 3 ms (mean ± s.e.m). The majority of units had less than 1% of short ISIs. **c,** Histogram of the mean firing rates over the 33 neurons recorded in the hippocampus. On average, neurons exhibited mean FRs of 4.53 ± 1.54 spikes/s (mean ± s.e.m). The firing rates of these neurons did not differ significantly between aversive scenes that were later remembered (FRs eR = 4.32 ± 1.40 spikes/s, mean ± s.e.m) or forgotten (FRs eKF = 4.36 ± 1.45 spikes/s, mean ± s.e.m). **d,** Waveform peak signal-to-noise ratio (SNR) for each neuron. On average, the SNR of the mean waveform peak was 6.50 ± 0.49 (mean ± s.e.m).

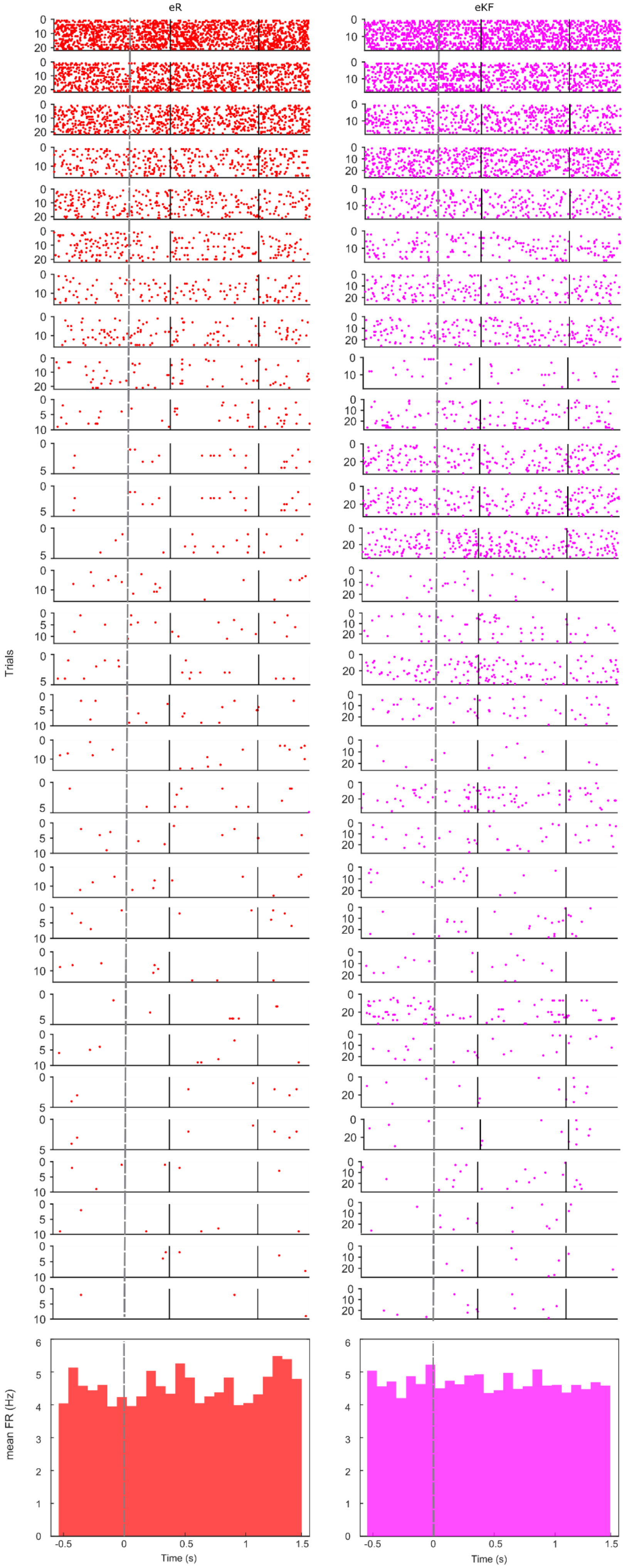

eR
eKF
Trials
mean FR (Hz)
Time (s)
Time (s)

Supplementary Fig. 22. Event-related raster plot showing hippocampal spikes over peri-stimulus time (x-axis) for eR (left column) and eKF trials (right column) for each unit (*n*=31). Units are sorted in descending order as a function of spike number across trials for eR condition. Dotted vertical line represent the stimulus onset. Black vertical lines represent the post stimulus time interval (from 0.41 to 1.1 s) on which PACOi and SFC analysis were performed. Bottom: histogram showing the mean firing rate for all units over time for eR and eKF conditions.

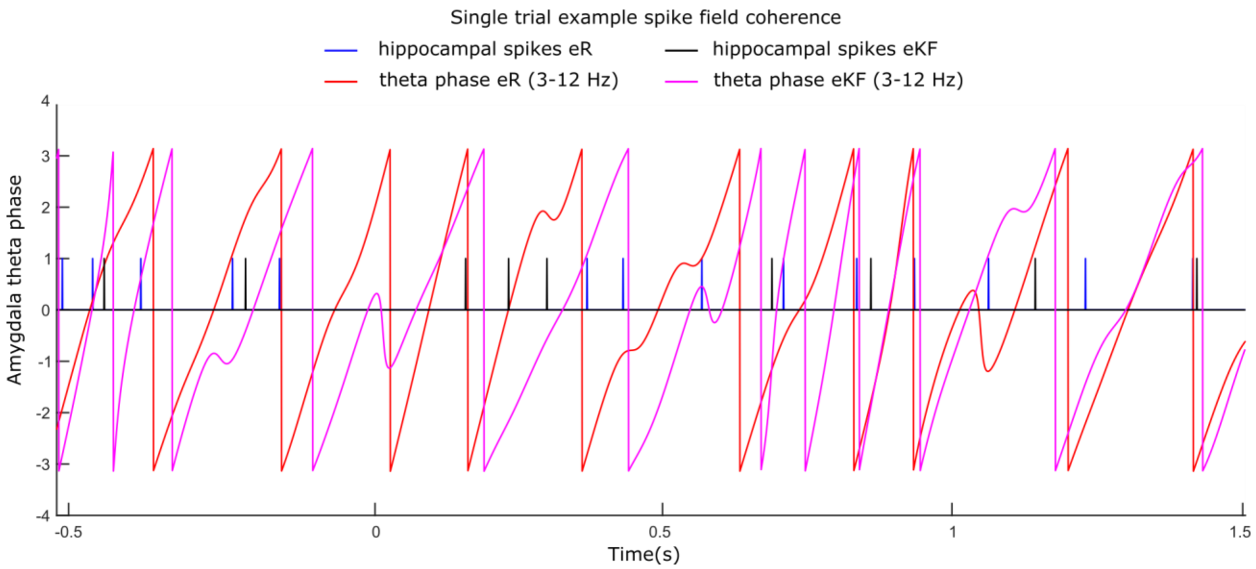

Supplementary Fig. 23. One example trial for eR and one example trial for eKF (patient Z5) showing the occurrence of hippocampal spikes from one unit in relation with the ipsilateral amygdala theta phase.

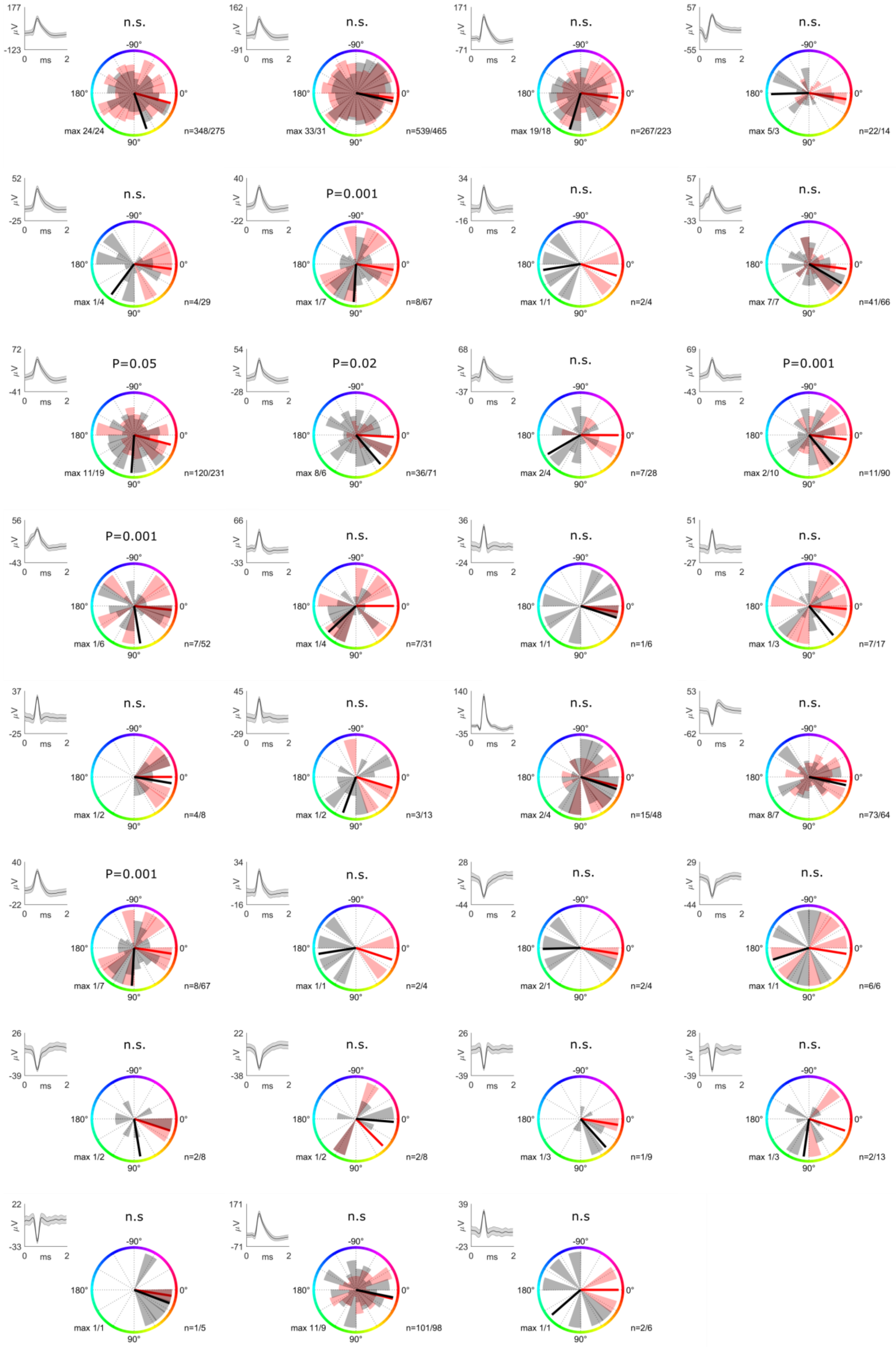
n.s.
max 24/24
n=348/275
n.s.
max 33/31
n=539/465
n.s.
max 19/18
n=267/223
n.s.
max 5/3
n=22/14
n.s.
max 1/4
n=4/29
P=0.001
max 1/7
n=8/67
n.s.
max 1/1
n=2/4
n.s.
max 7/7
n=41/66
P=0.05
max 11/19
n=120/231
P=0.02
max 8/6
n=36/71
n.s.
max 2/4
n=7/28
P=0.001
max 2/10
n=11/90
P=0.001
max 1/6
n=7/52
n.s.
max 1/4
n=7/31
n.s.
max 1/1
n=1/6
n.s.
max 1/3
n=7/17
n.s.
max 1/2
n=4/8
n.s.
max 1/2
n=3/13
n.s.
max 2/4
n=15/48
n.s.
max 8/7
n=73/64
P=0.001
max 1/7
n=8/67
n.s.
max 1/1
n=2/4
n.s.
max 2/1
n=2/4
n.s.
max 1/1
n=6/6
n.s.
max 1/2
n=2/8
n.s.
max 1/2
n=2/8
n.s.
max 1/3
n=1/9
n.s.
max 1/3
n=2/13
n.s
max 1/1
n=1/5
n.s
max 11/9
n=101/98
n.s
max 1/1
n=2/6

Supplementary Fig. 24. **Single neuron SFC amygdala theta hippocampus spikes *n*=31 (Cohort 2).** SFC phase opposition between eR and eKF trials for each observed neuron. Red and black shaded area represents spikes per phase bin (*n*=20) for each condition, red and black line is the realigned preferential phase for eR and eKF condition, respectively. *P* values from the circular Kuiper test (two-sided paired t-test) are reported on the top of each neuron showing significant phase opposition (n.s.: not significant); max is the maximum number of spikes occurring in a certain bin for each condition (eR/eKF); *n* is the number of total spikes per neuron observed from 0.4 to 1.1 s for each condition (eR/eKF). Upper left subpanel: single-neuron waveform (mean ± std).

Supplementary Table 1. Cohort 1 patient demographic and clinical data

Patient	Sex	Hand-edness	Age (range)	Age (years) at onset of epilepsy	Aetiology	Lesion location	Seizure type (frequency per months)	Drugs and dose (mg)	VIQ	PIQ	Education completed	% trials without epileptic spikes in amygdala	% trials without epileptic spikes in hippocampus
**Z1**	F	R	45-55	5	Hippocampal sclerosis	Left hippocampal sclerosis	Monthly CPS<br>Monthly SG TCS	OXC 1200<br>LEV 1500<br>CLB 15<br>LCS 200	75	98	Secondary	87.71 %	87.71 %
**02**	F	R	30-40	21	Hippocampal sclerosis plus focal dysplasia	Right hippocampal sclerosis plus porencephalic cyst over the parieto-occipital junction	Weekly CPS	LCS 400<br>LEV 3000	103	91	Tertiary	92.43 %	NA
**04**	F	R	18-28	12	Focal dysplasia	Extensive lesion over the left frontal region involving dorsolateral and orbitofrontal cortex and anterior border of the cingulum	Daily CPS<br>Daily SPS	CBZ 600<br>CNZ 3.5<br>LEV 1500<br>TOP 400	86	80	Tertiary	87.5 %	NA
**06**	M	R	45-55	14	Hippocampal sclerosis	Left hippocampal sclerosis	Weekly CPS<br>Monthly SG TCS	LGT 200<br>CLB 30<br>LCS 300	115	86	Tertiary	Left 98.3 % Right 100 %	Right 100 %
**13**	F	R	45-55	16	Focal dysplasia	Right basal temporal cortex	Monthly CPS	PGL 450<br>LCS 400	87	88	Secondary	77.3%	77.3%
**15**	F	R	30-40	16	Focal dysplasia	Left temporal pole	Daily CPS<br>Weekly SG TCS	OXC 1200<br>PHT 150	97	98	Tertiary	100 %	100 %
**16**	M	R	25-35	14	Reactive gliosis, diffuse microglia activation and small vessel vasculopathy	Medial wall of the left parietal region (precuneus and posterior cingulum)	Daily CPS<br>4 SG TCS yearly	LCS 400<br>ECZ 800<br>LTG 300<br>VAL 300<br>CLB 20<br>ESC 20	102	93	Tertiary	Left Right<br>94.06% 88.98%	Left Right<br>94.06% 88.98%
**21**	F	R	25-35	12	Periventricular heterotopia	Left occipital horn heterotopia	Weekly CPS	LEV 1000	104	87	Tertiary	Left 90% Right 94.11%	NA
**25**	M	R	25-35	13	Focal dysplasia	Right posterior temporobasal region	Weekly CPS<br>Monthly SG TCS	CBZ 1200<br>LEV 1000<br>LTG 200<br>ESC 10	116	97	Tertiary	79.16%	79.16%
**27**	M	R	25-35	21	Focal dysplasia	Left temporal pole	Weekly CPS<br>Monthly SG TCS	LGT 300<br>VPA 1000<br>Perampanel 8	69	102	Secondary	98.33%	NA
**32**	M	R	50-60	21	Encephalocele	Left temporal pole	Weekly CPS	LTG 500<br>LCM 400<br>LEV 3000	100	119	Tertiary	Left 100 % Right 100 %	Left<br>100%
**33**	F	R	18-28	14	Inflamatory lesion	Right parietal cortex	Weekly CPS<br>Montly SGTCS	ZNS 200<br>CBZ 600<br>LCM 450<br>CBZ 30	78	93		96.58%	NA
**34**	M		18-28	16	Postraumatic	Left temporo-occipital	Montly SGTCS<br>Montly CPS	CBZ 600<br>LCM 400<br>LEV 1500	86	81	Secondary	100%	NA

Abbreviations: CBZ=carbamazepine; CLB=clobazam; CNZ=clonazepam; CPS=complex partial seizure; ECZ=eslicarbazepine; ESC=escitalopram; LCS=lacosamide; LEV=levetiracetam; LTG=lamotrigine; NA=Not applicable; OXC=oxcarbazepine; PGL=pregabalin; PHT=phenytoin; PIQ/VIQ=Procedural/Verbal Intelligence Quotient of the Wechsler Adult Intelligence Scale, version III (WAIS-III); SG TCS=secondary generalized tonic clonic seizure; SPS=simple partial seizure; TOP=topiramate; VAL=valproate.; ZNS= zonisamide

31

Supplementary Table 2. Cohort 2 patient demographic, clinical data

Patient	Sex	Hand-edness	Age (range)	Aetiology	Lesion location
**Z1**	F	R	45-55	Hippocampal sclerosis	Left hippocampus
Z2	M	R	30-40	Focal cortical dysplasia	Right frontal cortex
Z5	M	R	30-40	Dysembryoplastic neuroepithelia tumor	Left anterior hippocampus
Z6	F	R	25-35	Unclear etiology	Unclear
Z8	F	R	25-35	Non-lesional	
Z10	M	R	50-60	Hippocampal sclerosis	Right hippocampus

Supplementary Table 3. Behavioral data from 18 patients (Cohorts 1 and 2). Raw trial number for emotionally aversive (e) and neutral (n) correctly remembered, remembered false alarms, correctly known and known false alarms (R, RFA, K, KFA, respectively) responses for all patients (*n*=18). Individual patient performance plotted in Fig. 1b is expressed in percentage calculated as number of items reported here divided by the total number of either emotional (40) or neutral (80) stimuli seen at encoding multiplied by 100.

Patient	Re	ReFas	eK	eKFas	nR	nRFas	nK	nKFas
**02**	7	1	21	15	10	0	36	14
**04**	20	3	5	7	40	13	19	22
**06**	10	1	13	13	11	3	25	9
**13**	27	21	1	3	47	16	9	10
**15**	19	10	7	7	19	9	16	19
**16**	19	12	8	9	23	11	21	22
**21**	24	11	6	9	48	9	12	14
**25**	16	3	15	13	25	6	29	25
**27**	17	13	14	8	32	14	9	13
**32**	25	10	8	15	38	14	19	26
**33**	21	10	12	8	20	1	12	8
**34**	21	15	11	17	4	3	21	15
**Z1**	24	24	6	5	22	16	8	16
**Z2**	4	2	0	0	9	4	1	0
**Z5**	15	7	13	13	24	6	18	16
**Z6**	12	1	5	8	33	7	16	14
**Z8**	4	4	18	21	12	3	23	11
**Z10**	9	10	1	1	3	2	2	1
**Mean**	16.3 (1.69)	8.77 (1.59)	9.11 (1.39)	9.55 (1.31)	23.33 (3.29)	7.61 (1.25)	16.44 (2.12)	14.16 (1.69)

Supplementary Table 4. Behavioral data from 18 patients (Cohorts 1 and 2). Reaction time at encoding. Single patient RT values, group mean ± s.e.m in ms. RTs are longer in response to aversive *vs*. neutral pictures (two-sided paired t-test T_{17} =3.66, *P*=0.002).

**Encoding**		
**Patient**	**Aversive**	**Neutral**
**02**	1587	1564
**04**	1543	1361
**06**	1236	1147
**13**	1237	1025
**15**	733	783
**16**	1615	1349
**21**	1437	1039
**25**	1315	1226
**27**	1610	1538
**32**	1500	1375
**33**	2254	1802
**34**	613	611
**Z1**	1253	1199
**Z2**	575	562
**Z5**	1628	1672
**Z6**	1012	973
**Z8**	1391	1244
**Z10**	1619	1529
**Mean**	1342.11 (97.41)	1222.16 (81.87)

Supplementary Table 5. Behavioral data from 18 patients (Cohorts 1 and 2). Reaction time at recognition for all memory categories: remember (R), remember false alarm (RFA), know (K), know false alarm (KFA), miss (Miss) and correct rejected (Crj) for emotional (e) and neutral (n) trials. Single patient RT values, group mean and s.e.m in ms. We compared RTs of remembered (R) items to the RTs of known (K) and correct rejection (Crj) responses for emotional (eR, eK, Crje) and neutral items (nR, nK, Crjn). The ANOVA revealed a significant main effect of memory $F_{(2, 16)}$=8.85, P=0.003, but no main effect of emotion $F_{(1, 17)}$=0.48, P=0.49 or emotion by memory interaction $F_{(2, 16)}$=2.87, P=0.086, η^2=0.26.

**Recognition**						
**Patient**	**Re**	**R FAe**	**Ke**	**K FAe**	**Misse**	**Crje**
**02**	1562	1858	1787	1859	1776	1737
**04**	1647	1566	2348	1833	2057	1855
**06**	1381	2160	1697	2000	1426	1432
**13**	1368	1259	1735	2219	1772	1611
**15**	1524	1462	1702	1554	1868	1859
**16**	1587	1845	2125	2454	2208	1700
**21**	1453	1269	1845	2502	2066	1850
**25**	1957	1899	2203	2565	1781	2129
**27**	1546	1392	2146	2377	2390	2398
**32**	1483	1597	1770	1788	1880	1690
**33**	1482	1487	1927	1653	1902	1421
**34**	698	701	975	907	925	839
**Z1**	1283	1475	2108	2317	1831	1949
**Z2**	554	598	--	--	549	506
**Z5**	1684	1543	2095	2316	1976	2042
**Z6**	1378	1687	1391	1461	1186	1293
**Z8**	1518	1834	1726	1717	1695	1781
**Z10**	1567	1577	3188	2245	1566	1343
**Mean**	1426.22 (77.11)	1511.61 (91.91)	1927.52 (108.92)	1986.29 (105.44)	1714.11 (106.74)	1635.27 (106.52)

	**Rn**	**R FAn**	**Kn**	**K FAn**	**Missn**	**Crjn**
**02**	1592	--	1749	1797	1539	1454
**04**	1527	1887	1947	2113	1622	1742
**06**	1436	1986	1783	1758	1183	1185
**13**	1792	1509	1885	2026	1450	1464
**15**	1643	1501	1473	1518	1579	1499
**16**	1665	2303	2557	2690	1836	1881
**21**	1186	1095	1769	1897	1510	1617
**25**	2104	2009	2275	2397	1827	1884
**27**	1686	2035	2136	2273	2415	1975
**32**	1420	1721	1814	1882	1649	1651
**33**	1573	1465	1755	1393	1159	1055

**34**	759	715	894	805	760	800
**Z1**	3116	1442	1803	1921	1831	1949
**Z2**	583	590	834	--	546	539
**Z5**	1881	1708	2131	2339	2062	1920
**Z6**	1185	1223	1571	1495	1293	1252
**Z8**	1632	2468	1766	1645	1638	1485
**Z10**	1134	2096	1663	3095	1356	1197
**Mean**	1550.77 (127.91)	16325.29 (122.92)	1766.94 (98.51)	1943.76 (125.30)	1499.22 (101.96)	1449.88 (92.39)

Supplementary Table 6. Total number of trials per patient and condition (Cohort 1). r, right electrode in the case of bilateral electrodes.

Patient	eR aversive remember	eK aversive known	eF aversive forgotten	nR neutral remember	nK neutral known	nF neutral forgotten
06r	10	13	17	11	25	43
13	19	7	14	19	16	44
15	19	8	13	23	21	36
16	24	6	10	48	12	18
16r	24	6	10	48	12	18
25	17	14	9	32	9	39
32	12	14	14	39	18	23
33	21	12	5	20	12	47
Z1r	21	11	6	4	21	51

Supplementary Table 7. Number of analyzed trials per patient and conditions following exclusions based on both time- and frequency-domain artifact rejection, and random selection used for the connectivity analysis (Cohort 1). NA, not applicable (data from patient Z1 were not used in testing for emotion by memory interaction due to low number of nR trials).

Patient	eR aversive remember	eKF aversive known/forgotten	nR neutral remember	nKF neutral known/forgotten	Random selection of trials
06r	10	29	11	68	10
13	14	14	14	35	14
15	19	19	22	57	19
16	21	16	43	26	16
16r	19	11	33	22	11
25	14	9	15	26	9
32	12	28	39	41	12
33	14	10	16	37	10
Z1r	16	11	2	52	2/NA

Supplementary note 1. In the present study statistical inference was based on a fixed-effects approach. The repeated measure is amygdala (*n*=17, 9 unilateral and 4 bilateral electrodes) or hippocampus (*n*=9, 7 unilateral electrodes and 1 bilateral electrode), and amygdala-hippocampus pairs (*n*=9, 7 unilateral electrodes and 1 bilateral electrode). To complement the fixed effects approach, we ran a hierarchical linear model analysis.

For this purpose, we used a random intercept model plus addition of Level 1 predictors following the formula Yij=γ00+ μ0j+ γsessions + eij. The dependent variable is the mean gamma power extracted from the significant clusters obtained from time-frequency analyses in the amygdala and hippocampus, respectively. Level 1 predictors pertain to patients that correspond to different recordings or to the same patient when bilateral electrodes are considered. We found that amygdala and hippocampal responses were unrelated to recorded patient (mean gamma difference in the amygdala between (eR - eKF) and (nR - nKF), $F_{(1, 14)}$=2.4, *P*=0.14 Fig. 2c;); mean gamma difference in the hippocampus (R-KF), $F_{(1, 7)}$=0.27, *P*=0.61 , Fig. 2f). These findings are in line with a fixed effects assumption, where the dependent variable is presumably constant across the units of observation. Note that only one patient with bilateral recordings contributed to the connectivity statistics where amygdala-hippocampus pairs are used. Seizure onset was localized to the left precuneus and posterior cingulate in this patient, and not to the medial temporal lobe.

Supplementary References

1 Vinck, M., van Wingerden, M., Womelsdorf, T., Fries, P. & Pennartz, C. M. A. The pairwise phase consistency: A bias-free measure of rhythmic neuronal synchronization. *NeuroImage* **51**, 112-122, doi:10.1016/j.neuroimage.2010.01.073 (2010).
