# Supplementary material for: Aversive memory formation in humans involves an amygdala-hippocampus phase code
Source: Nat Commun. 2022 Oct 27;13:6403. doi: 10.1038/s41467-022-33828-2 (PMC9613775; doi:10.1038/s41467-022-33828-2)
Supplement: Supplementary file 3 — Reporting Summary [file 41467_2022_33828_MOESM3_ESM.pdf]

nature portfolio

Corresponding author(s): Manuela Costa, Bryan Strange

Last updated by author(s): Sep 4, 2022

Reporting Summary

Nature Portfolio wishes to improve the reproducibility of the work that we publish. This form provides structure for consistency and transparency in reporting. For further information on Nature Portfolio policies, see our Editorial Policies and the Editorial Policy Checklist.

Statistics

For all statistical analyses, confirm that the following items are present in the figure legend, table legend, main text, or Methods section.

n/a	Confirmed	
☐	☒	The exact sample size (n) for each experimental group/condition, given as a discrete number and unit of measurement
☐	☒	A statement on whether measurements were taken from distinct samples or whether the same sample was measured repeatedly
☐	☒	The statistical test(s) used AND whether they are one- or two-sided<br>*Only common tests should be described solely by name; describe more complex techniques in the Methods section.*
☒	☐	A description of all covariates tested
☐	☒	A description of any assumptions or corrections, such as tests of normality and adjustment for multiple comparisons
☐	☒	A full description of the statistical parameters including central tendency (e.g. means) or other basic estimates (e.g. regression coefficient) AND variation (e.g. standard deviation) or associated estimates of uncertainty (e.g. confidence intervals)
☐	☒	For null hypothesis testing, the test statistic (e.g. F, t, r) with confidence intervals, effect sizes, degrees of freedom and P value noted<br>*Give P values as exact values whenever suitable.*
☒	☐	For Bayesian analysis, information on the choice of priors and Markov chain Monte Carlo settings
☒	☐	For hierarchical and complex designs, identification of the appropriate level for tests and full reporting of outcomes
☐	☒	Estimates of effect sizes (e.g. Cohen's d, Pearson's r), indicating how they were calculated

Our web collection on statistics for biologists contains articles on many of the points above.

Software and code

Policy information about availability of computer code

Data collection	At the Ruber Hospital Internacional, Madrid (Cohort 1), ongoing intracranial EEG (iEEG) activity was acquired using an XLTEK EMU128FS amplifier (XLTEK, Oakville, Ontario, Canada). Intracranial data in Zurich (Cohort 2) were acquired with a Neuralynx ATLAS syste .
Data analysis	Custom code written in MATLAB version R2017b (the Mathworks, Natick, MA, USA) and Fieldtrip functons (https://www.fieldtriptoolbox.org) were used to analyze all data (Fieldtrip Toolbox 20190203 and 20210212). Anatomical data analysis was performed using FreeSurfer version 6, https://surfer.nmr.mgh.harvard.edu/. Segmentation of hippocampal subfields and amygdala nuclei was performed using FreeSurfer version 7.2.0. Electrode contact locations are displayed using Paraview (www.paraview.org).

For manuscripts utilizing custom algorithms or software that are central to the research but not yet described in published literature, software must be made available to editors and reviewers. We strongly encourage code deposition in a community repository (e.g. GitHub). See the Nature Portfolio guidelines for submitting code & software for further information.

Data

Policy information about availability of data

All manuscripts must include a data availability statement. This statement should provide the following information, where applicable:

- Accession codes, unique identifiers, or web links for publicly available datasets
- A description of any restrictions on data availability
- For clinical datasets or third party data, please ensure that the statement adheres to our policy

Data availability. All data needed to evaluate the conclusions in the paper are present in the paper and/or the supplementary materials. The datasets generated during and/or analysed during the current study are available in the CostaLozanoetal repository, https://github.com/TheStrangeLab/CostaLozanoetal. The raw data

will be made avaiable upon reasonable request.

Field-specific reporting

Please select the one below that is the best fit for your research. If you are not sure, read the appropriate sections before making your selection.

☒ Life sciences ☐ Behavioural & social sciences ☐ Ecological, evolutionary & environmental sciences

For a reference copy of the document with all sections, see nature.com/documents/nr-reporting-summary-flat.pdf

Life sciences study design

All studies must disclose on these points even when the disclosure is negative.

Sample size	No statistical methods were used to pre-determine sample sizes but our sample sizes are larger or equal to those reported in previous publications (Zheng et al, 2017; Mendez-Bertolo et al 2016).
Data exclusions	We analyzed electrophysiological responses from 17 amygdalae from 13 patients (five had left, 4 right, and 4 bilateral medial temporal electrodes in the amygdala). Eleven patients also had electrodes in the ipsilateral anterior hippocampus. The hippocampal recording of one patient was excluded after hippocampal sclerosis was reported on the same side as the unilateral electrode implantation. For the second patient showing hippocampal sclerosis, only the non-pathological side was included. One further patient did not meet our criteria for spike-free trials (75%). We therefore analyzed electrophysiological responses from 9 hippocampi from 8 patients (1 patient had bilateral hippocampal electrodes).
Replication	For all connectivity measures we restricted the analysis to patients with electrodes in both amygdala and the hippocampus. All patients in Cohort1 have electrodes implanted in the amygdala, we thus repeated the time frequency analysis restricting the group of patients to only those with electrodes in both structures and we found similar amygdala gamma responses.
Randomization	Encoding and recognition sessions after 24h were conducted during the third and fourth post-operative days for all patients in Madrid, and on the second and third post-operative days for all patients recorded in Zurich. Our design is a within-subject analysis: all patients viewd all trials. Aversive and neutral pictures were presented pseudo-randomly with a constraint that aversive scenes were separeted by at least one neutral scene
Blinding	As participants were not allocated into different experimental groups, blinding was not relevant for data collection.<br>Participants were not aware of the study's goal.

Reporting for specific materials, systems and methods

We require information from authors about some types of materials, experimental systems and methods used in many studies. Here, indicate whether each material, system or method listed is relevant to your study. If you are not sure if a list item applies to your research, read the appropriate section before selecting a response.

Materials & experimental systems

n/a	Involved in the study	
☒	☐	Antibodies
☒	☐	Eukaryotic cell lines
☒	☐	Palaeontology and archaeology
☒	☐	Animals and other organisms
☐	☒	Human research participants
☒	☐	Clinical data
☒	☐	Dual use research of concern

Methods

n/a	Involved in the study	
☒	☐	ChIP-seq
☒	☐	Flow cytometry
☐	☒	MRI-based neuroimaging

Human research participants

Policy information about studies involving human research participants

Population characteristics	Cohort1: Participants were 13 medication-resistant presurgical epilepsy patients with depth electrodes surgically implanted to aid seizure focus localization, age 18-59 years, 7 females.<br>Cohort 2: This included 6 medication-resistant presurgical epilepsy patients implanted in the medial temporal lobe for diagnostic purposes, age 29-56 years, 3 females.
Recruitment	We included in the study all patients that accepted to participate in the study and that had electrodes implantation in the amygdala and/or hippocampus. Implantation sites were chosen solely on the basis of clinical criteria.
Ethics oversight	All patients signed informed consent. The study had full approval from the local ethics committees of the Hospital Ruber Internacional, Madrid, Spain and Kantonale Ethikkommission, Zurich, Switzerland (PB-2016-02055).

Note that full information on the approval of the study protocol must also be provided in the manuscript.

Magnetic resonance imaging

Experimental design

Design type	Clinical protocol for structural scanning
Design specifications	We used structural scanning only for electrode contact localization
Behavioral performance measures	We did not record any behavioural measures as the structural scanning was used only for contact localization.

Acquisition

Imaging type(s)	structural MRI, CT
Field strength	MRIs were acquired on a 3T
Sequence & imaging parameters	T1-weighted MRI
Area of acquisition	whole brain scanning

Diffusion MRI ☐ Used ☒ Not used

Preprocessing

Preprocessing software	SPM8 (http://www.fil.ion.ucl.ac.uk/spm).
Normalization	Data were normalized to MNI space
Normalization template	MNI
Noise and artifact removal	For each patient, the post-electrode placement CTs (post-CT) was co-registered to the pre-electrode placement T1-weighted magnetic resonance images (pre-MRI). To optimize co-registration, both brain images were first skull-stripped. For CTs this was done by filtering out all voxels with signal intensities between 100 and 1300 HU.
Volume censoring	As we only used structural MRI, volume censoring was not used.

Statistical modeling & inference

Model type and settings	Patients did not undertake a behavioural study. The structural scanning was used for electrode contact localization.
Effect(s) tested	No effects were tested.

Specify type of analysis: ☐ Whole brain ☐ ROI-based ☐ Both

Statistic type for inference (See Eklund et al. 2016)	We did not run any statistical test.
Correction	We did not apply statistical corrections.

Models & analysis

n/a	Involved in the study
☒	☐ Functional and/or effective connectivity
☒	☐ Graph analysis
☒	☐ Multivariate modeling or predictive analysis
